# Characterization of the wheat VQ protein family and expression of candidate genes associated with seed dormancy and germination

**DOI:** 10.1186/s12870-022-03430-1

**Published:** 2022-03-15

**Authors:** Xinran Cheng, Chang Gao, Xue Liu, Dongmei Xu, Xu Pan, Wei Gao, Shengnan Yan, Hui Yao, Jiajia Cao, Xiaoyu Min, Jie Lu, Cheng Chang, Haiping Zhang, Chuanxi Ma

**Affiliations:** 1 College of Agronomy, Key Laboratory of Wheat Biology and Genetic Improvement On Southern Yellow & Huai River Valley, Ministry of Agriculture and Rural Affairs, Anhui Agricultural University, Hefei, 230036 Anhui China; 2 National Key Laboratory for Crop Genetics and Germplasm Enhancement, Jiangsu Plant Gene Engineering Research Center, Nanjing Agricultural University, Nanjing, 210095 China

**Keywords:** Wheat, VQ Protein Family, Evolutionary Analysis

## Abstract

**Background:**

Seed dormancy and germination determine wheat resistance to pre-harvest sprouting and thereby affect grain yield and quality. *Arabidopsis VQ* genes have been shown to influence seed germination; however, the functions of wheat *VQ* genes have not been characterized.

**Results:**

We identified 65 *TaVQ* genes in common wheat and named them *TaVQ1–65*. We identified 48 paralogous pairs, 37 of which had Ka/Ks values greater than 1, suggesting that most *TaVQ* genes have experienced positive selection. Chromosome locations, gene structures, promoter element analysis, and gene ontology annotations of the *TaVQ*s showed that their structures determined their functions and that structural changes reflected functional diversity. Transcriptome-based expression analysis of 62 *TaVQ* genes and microarray analysis of 11 *TaVQ* genes indicated that they played important roles in diverse biological processes. We compared *TaVQ* gene expression and seed germination index values among wheat varieties with contrasting seed dormancy and germination phenotypes and identified 21 *TaVQ* genes that may be involved in seed dormancy and germination.

**Conclusions:**

Sixty-five TaVQ proteins were identified for the first time in common wheat, and bioinformatics analyses were used to investigate their phylogenetic relationships and evolutionary divergence. qRT-PCR data showed that 21 *TaVQ* candidate genes were potentially involved in seed dormancy and germination. These findings provide useful information for further cloning and functional analysis of *TaVQ* genes and introduce useful candidate genes for the improvement of PHS resistance in wheat.

**Supplementary Information:**

The online version contains supplementary material available at 10.1186/s12870-022-03430-1.

## Background

Wheat is a widely cultivated gramineous plant and one of the three most important cereals in the world [1]. It is a heterologous hexaploid derived from three closely related ancestors that have undergone two rounds of natural hybridization. Therefore, the large and complex genome of wheat (17 Gb) poses a significant challenge for wheat genome research [2, 3]. The completion of a whole genome sequence for wheat based on single chromosome sequencing has laid the foundation for wheat genomics research and wheat gene family identification.

Valine-glutamine (VQ) proteins are a class of plant-specific proteins with five highly conserved amino acids in the core FxxxVQxLTG sequence of the VQ motif [4], in which x represents any amino acid (aa) and VQ is a highly conserved pair of aa residues. Research on the VQ proteins has shown that the last three amino acids in almost all species are LTG, although some species have other variants, including FTG, ITG, LTA, and VTG [5]. In some VQ proteins of Gramineae species such as rice, maize, and Moso bamboo, VQ has mutated to VH in the conserved domain [5, 6]. VQ proteins are generally less than 300 aa in length and contain no or few introns [7]. To date, 34, 40, 61, 18, and 74 VQ proteins have been identified in *Arabidopsis*, rice, maize, grape, and soybean, respectively [6, 8–11]. According to bioinformatics predictions and experimental verification, some *Arabidopsis* VQ proteins are located in the nucleus, some in the plastid, and a few partly in the mitochondria [12].

VQ proteins play important roles in the regulation of plant growth and development and the response to abiotic and biotic stress [5–7, 13–17]. For instance, *AtCaMBP25* (*AtVQ15*) negatively regulates osmotic stress response during the early stages of seed germination and growth in *Arabidopsis* [13]. Likewise, *AtVQ9* expression responds strongly to NaCl treatment, and its mutation enhances salt stress tolerance in *Arabidopsis* [16]. *VQ54* and *VQ19* in maize, as well as *VQ2, VQ16*, and *VQ20* in rice, are highly expressed under drought induction [6, 11]. Soybean *VQ6* and *VQ53* are highly expressed in roots and stems under low nitrogen conditions [10]. *SIB1* (Sigma factor binding protein 1, also known as *AtVQ23*) was the first VQ motif protein discovered in *Arabidopsis* and participates in plant disease resistance signaling pathways [15]. *AtVQ21 (MSK1)* transgenic plants show enhanced resistance to the pathogen *Pseudomonas syringae* but reduced resistance to *Botrytis cinerea* [13, 18]. *AtVQ22* negatively regulates JA-mediated disease resistance signaling pathways [19], and rice *VQ22* shows high expression levels after rice blast infection [5]. *AtVQ14* (*IKU1*) participates in the regulation of endosperm development, thereby affecting the size of *Arabidopsis* seeds [20]. *AtVQ29* is involved in the photomorphogenesis of *Arabidopsis* seedlings and flowering time regulation [9]. In addition, the growth of *VQ17*, *VQ18*, *VQ8*, and *VQ22* transgenic *Arabidopsis* plants is inhibited, indicating that these genes play crucial roles in plant growth and development [8].

VQ proteins came to the attention of researchers because of their interactions with WRKY transcription factors, which are involved in regulating the plant’s defense response system [13, 15]. WRKY transcription factors belong to a large gene family and are ubiquitous in plants. Studies have shown that WRKY transcription factors are widely involved in plant growth and development and in resistance to adverse conditions [21–38]. For example, *AtVQ14* and *AtWRKY10* interact to form a protein complex that affects seed size in *Arabidopsis* [20]. *AtVQ15* and *AtWRKY25* interact and participate in high salt and osmotic stress response [13]. The interaction between *AtVQ22* and *AtWRKY28* negatively regulates JA-mediated disease resistance signaling pathways [19]. *AtVQ23 (SIB1)* and *AtVQ16 (SIB2)* interact with *WRKY33* to enhance the binding capacity of *WRKY33* to the W-box, thereby regulating plant disease resistance [6, 15]. *AtVQ21* can form ternary complexes with *AtWRKY33* and *AtMPK4* to regulate plant growth and disease resistance [4, 39]. In brief, VQ proteins are transcriptional regulatory cofactors that participate in growth and developmental processes and stress resistance through their interactions with transcription factors. However, until now, the VQ gene family has not been characterized in common wheat.

Pre-harvest sprouting (PHS) refers to the germination of wheat seeds within the spike of the mother plant that occurs in rainy or high moisture conditions before harvest. A series of physiological and biochemical reactions take place in wheat grains when PHS occurs. The activity of hydrolases such as amylase and proteolytic enzymes is enhanced, leading to starch and protein degradation and seriously affecting wheat processing quality and utilization value. In the international wheat market, when the germination rate of commercial wheat reaches 5%, it is regarded as feed wheat and its price is reduced, causing serious economic losses to producers [40, 41]. Seed dormancy and germination traits determine wheat PHS resistance: wheat varieties with higher levels of dormancy or lower germination percentages show higher resistance to PHS. Therefore, the identification of candidate genes that control seed dormancy and germination may help to reduce yield and quality losses caused by PHS. Previously, in *Arabidopsis, VQ18* and *VQ26* were found to be involved in seed germination via the ABA signaling pathway [42]. However, the functions of *VQ* genes in common wheat are largely unknown.

The objectives of this study were to identify *TaVQ* genes and to perform bioinformatics analysis, including phylogenetic tree construction and characterization of gene structures, conserved domains, chromosome positions, expression patterns, and promoter elements. In addition, we measured the expression levels of *TaVQ* genes in wheat varieties with contrasting seed dormancy and germination phenotypes by qRT-PCR to identify *TaVQ* gene family members that were potentially involved in seed dormancy and germination.

## Results

### Identification and attribute analysis of *VQ* candidate genes in wheat

A total of 65 *TaVQ* genes were identified, mapped to wheat chromosomes, and named *TaVQ1–TaVQ65*. The length of the encoded proteins ranged from 127 to 723 aa, with an average length of 220 aa. Their MWs ranged from 13,377.16 Da (*TaVQ52*) to 61,926.76 Da (*TaVQ1*). Information on chromosome positions, ORF lengths, and exon numbers is provided in Table 1. The majority of genes included one exon, and only four genes (*TaVQ13/-17/-18/-53*) contained two exons.

**Table 1:** Detailed information about the predicted TaVQ genes

Name	Gene ID	Location	ORF length(bp)	Size (aa)	MW(Da)	pI	Exons
TaVQ1	TraesCS1A02G338700	Chr1A:528,102,140–528,103,155	2172	723	61,926.76	4.99	1
TaVQ2	TraesCS2A01G487800	Chr2A:722,518,069–722,518,557	489	162	16,310.14	8.11	1
TaVQ3	TraesCS2A01G517000	Chr2A:740,346,569–740,347,388	636	211	22,232.87	9.77	1
TaVQ4	TraesCS2B01G314600	Chr2B:449,669,843–449,671,002	990	329	34,759.36	9.97	1
TaVQ5	TraesCS2B01G515400	Chr2B:710,280,174–710,280,671	498	165	16,828.73	6.09	1
TaVQ6	TraesCS2B01G545500	Chr2B:742,454,459–742,455,094	636	211	22,348.05	9.87	1
TaVQ7	TraesCS2D01G296200	Chr2D:378,525,321–378,526,310	990	329	34,841.5	9.97	1
TaVQ8	TraesCS2D01G488100	Chr2D:587,696,143–587,696,631	489	162	16,649.49	6.92	1
TaVQ9	TraesCS2D01G518600	Chr2D:608,745,741–608,746,370	630	209	22,044.73	9.93	1
TaVQ10	TraesCS3A01G044200	Chr3A:23,866,681–23,867,710	771	256	26,516.25	6.49	1
TaVQ11	TraesCS3A01G190200	Chr3A:235,380,871–235,382,316	702	233	24,220.28	6.11	1
TaVQ12	TraesCS3A01G334400	Chr3A:580,608,595–580,609,062	468	155	15,944.31	5.77	1
TaVQ13	TraesCS3B01G197900	Chr3B:225,257,528–225,258,102	486	161	17,544.85	5.7	2
TaVQ14	TraesCS3B01G219100	Chr3B:262,901,818–262,902,606	789	262	27,481.07	9.16	1
TaVQ15	TraesCS3B01G365300	Chr3B:577,490,273–577,490,731	459	152	15,724.13	6.07	1
TaVQ16	TraesCS3B01G478300	Chr3B:726,621,717–726,622,121	405	134	14,557.72	10.15	1
TaVQ17	TraesCS3D01G036600	Chr3D:13,254,563–13,257,748	774	257	26,587.4	6.7	2
TaVQ18	TraesCS3D01G174600	Chr3D:156,240,964–156,246,686	489	162	17,816.12	5.45	2
TaVQ19	TraesCS3D01G193700	Chr3D:184,379,368–184,380,834	717	238	24,770.88	6.12	1
TaVQ20	TraesCS3D02G272900	Chr3D:378,529,151–378,530,444	2154	717	61,510.13	5	1
TaVQ21	TraesCS4A01G369400	Chr4A:641,502,472–641,502,912	441	146	14,995.88	11.33	1
TaVQ22	TraesCS4A01G411100	Chr4A:683,787,345–683,787,800	456	151	15,683.79	10.54	1
TaVQ23	TraesCS4A01G411200	Chr4A:683,800,842–683,801,246	405	134	14,461.68	10.61	1
TaVQ24	TraesCS4A01G411300	Chr4A:683,805,840–683,806,277	438	145	15,124.32	10.9	1
TaVQ25	TraesCS4B01G178500	Chr4B:391,157,880–391,158,956	438	145	14,849.46	7.1	1
TaVQ26	TraesCS4B01G311200	Chr4B:601,276,598–601,277,606	435	144	14,860.96	11.14	1
TaVQ27	TraesCS4B01G311300	Chr4B:601,284,457–601,284,921	465	154	16,000.23	10.55	1
TaVQ28	TraesCS4B01G311400	Chr4B:601,306,252–601,306,716	465	154	16,004.18	10.55	1
TaVQ29	TraesCS4D01G180100	Chr4D:313,885,547–313,886,431	450	149	15,190.79	8.01	1
TaVQ30	TraesCS4D02G021000	Chr4D:8,997,650–8,998,244	1002	333	26,481.38	5.19	1
TaVQ31	TraesCS5A01G010900	Chr5A:7,167,768–7,168,166	399	132	14,359.52	10.15	1
TaVQ32	TraesCS5A01G027100	Chr5A:22,485,164–22,486,133	759	252	26,758.28	9.44	1
TaVQ33	TraesCS5A01G189700	Chr5A:393,252,902–393,254,173	1272	423	42,945.76	6.55	1
TaVQ34	TraesCS5A01G427000	Chr5A:611,817,416–611,818,023	444	147	15,224.72	9.56	1
TaVQ35	TraesCS5B01G008700	Chr5B:9,243,595–9,244,008	414	137	14,803.08	9.88	1
TaVQ36	TraesCS5B01G026300	Chr5B:25,061,684–25,062,761	732	243	25,550.96	9.51	1
TaVQ37	TraesCS5B01G193500	Chr5B:349,712,051–349,713,313	1263	420	42,784.59	6.37	1
TaVQ38	TraesCS5B01G428900	Chr5B:604,226,071–604,227,208	444	147	15,152.49	9.56	1
TaVQ39	TraesCS5B01G503300	Chr5B:669,909,164–669,909,637	474	157	16,299.42	11.29	1
TaVQ40	TraesCS5D01G015700	Chr5D:9,068,493–9,069,444	399	132	14,358.51	10.15	1
TaVQ41	TraesCS5D01G034800	Chr5D:34,043,996–34,045,414	735	244	25,783.27	9.09	1
TaVQ42	TraesCS5D01G201200	Chr5D:304,876,446–304,878,167	1722	573	59,359.24	10.4	1
TaVQ43	TraesCS5D01G434900	Chr5D:490,166,006–490,166,449	444	147	15,110.47	9.56	1
TaVQ44	TraesCS6A01G156200	Chr6A:145,133,789–145,134,454	666	221	22,431.63	9.85	1
TaVQ45	TraesCS6A01G300700	Chr6A:533,466,786–533,467,235	450	149	15,970.96	10.16	1
TaVQ46	TraesCS6A01G301100	Chr6A:534,747,170–534,747,685	516	171	17,845.92	9.49	1
TaVQ47	TraesCS6B01G184400	Chr6B:207,154,528–207,155,778	681	226	23,005.3	10.08	1
TaVQ48	TraesCS6B01G330400	Chr6B:579,549,916–579,550,626	492	163	17,065	9.2	1
TaVQ49	TraesCS6D01G146200	Chr6D:116,952,516–116,953,190	675	224	22,649.97	9.85	1
TaVQ50	TraesCS6D01G280600	Chr6D:388,136,875–388,137,396	522	173	18,231.26	9.2	1
TaVQ51	TraesCS7A01G326900	Chr7A:475,165,941–475,166,708	768	255	26,443.91	10.07	1
TaVQ52	TraesCS7A01G349400	Chr7A:512,169,425–512,169,814	390	129	13,377.16	9.45	1
TaVQ53	TraesCS7A01G375900	Chr7A:549,001,358–549,001,981	603	200	21,229.86	10.61	2
TaVQ54	TraesCS7A01G401900	Chr7A:581,344,730–581,345,350	621	206	22,373.4	6.07	1
TaVQ55	TraesCS7A01G478700	Chr7A:671,483,204–671,483,848	645	214	21,698.87	9.51	1
TaVQ56	TraesCS7B01G227600	Chr7B:427,821,922–427,822,689	768	255	26,484.95	9.77	1
TaVQ57	TraesCS7B01G233400	Chr7B:436,838,688–436,839,071	384	127	13,522.39	8.91	1
TaVQ58	TraesCS7B01G277500	Chr7B:507,836,057–507,836,671	615	204	21,621.29	10.39	1
TaVQ59	TraesCS7B01G302000	Chr7B:538,864,067–538,864,690	624	207	22,455.42	5.97	1
TaVQ60	TraesCS7B01G381400	Chr7B:646,209,297–646,210,514	627	208	21,214.45	9.51	1
TaVQ61	TraesCS7D01G323700	Chr7D:413,441,710–413,442,474	765	254	26,249.67	9.63	1
TaVQ62	TraesCS7D01G329300	Chr7D:420,782,426–420,782,812	387	128	13,671.46	8.93	1
TaVQ63	TraesCS7D01G372400	Chr7D:481,836,636–481,837,615	477	158	16,567.59	10.19	1
TaVQ64	TraesCS7D01G395700	Chr7D:510,450,077–510,450,709	636	211	22,939.95	5.67	1
TaVQ65	TraesCS7D01G466100	Chr7D:579,961,244–579,961,867	624	207	21,039.19	9.3	1

### Phylogenetic trees of VQ proteins from wheat, maize, poplar, rice, and *Arabidopsis*

**Fig. 1:**
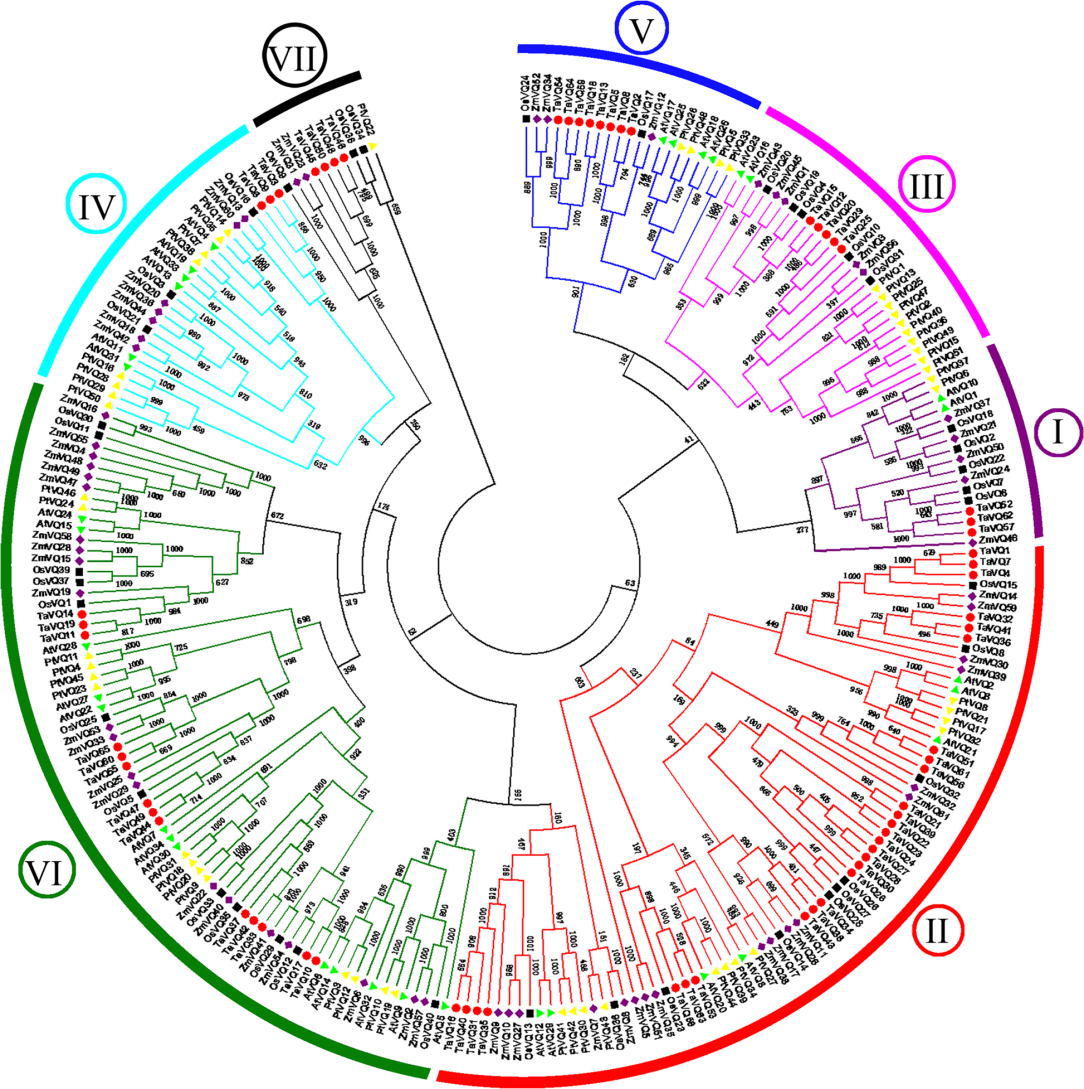
Phylogeny of VQ proteins from wheat, rice, poplar, maize, and *Arabidopsis*. The 65 *TaVQ* proteins, 40 *OsVQ* proteins, 51PtVQ proteins, 61 *ZmVQ* proteins, and 34*AtVQ* proteins are clustered into seven subfamilies. Details of VQ genes from *Arabidopsis*, maize, poplar and rice are listed in Table S1. The red circle, black square, yellow triangle, purple diamond and green inverted triangle represent the VQ gene families of wheat, rice, poplar, maize and *Arabidopsis* respectively. Different colors of inner ring and outer ring represent different subfamilies

To explore the evolutionary relationships among *VQ* genes from wheat, *Arabidopsis*, rice, poplar, and maize, we downloaded published VQ protein sequences from these species (Table S1) [8–10] and constructed a phylogenetic tree (Fig. 1). Based on the original division and naming of *VQ* subfamilies in *Arabidopsis* and rice, we divided the 251 *VQ* genes (34 *AtVQ* genes, 40 *OsVQ* genes, 51 *PtVQ* genes, 61 *ZmVQ* genes, and 65 *TaVQ* genes) into seven subfamilies (VQI, VQII, VQIII, VQIV, VQV, VQVI, and VQVII). The VQII subfamily contained the largest number of genes (28) (Fig. 1). Members of the VQ family from rice, maize, and wheat, which belong to the Gramineae, were interspersed, whereas those of *Arabidopsis*, a model plant from the Cruciferae, formed separate clades, probably due to the relatively distant relationship between monocots and dicots.

### Structural analysis of the *VQ* gene family

**Fig. 2:**
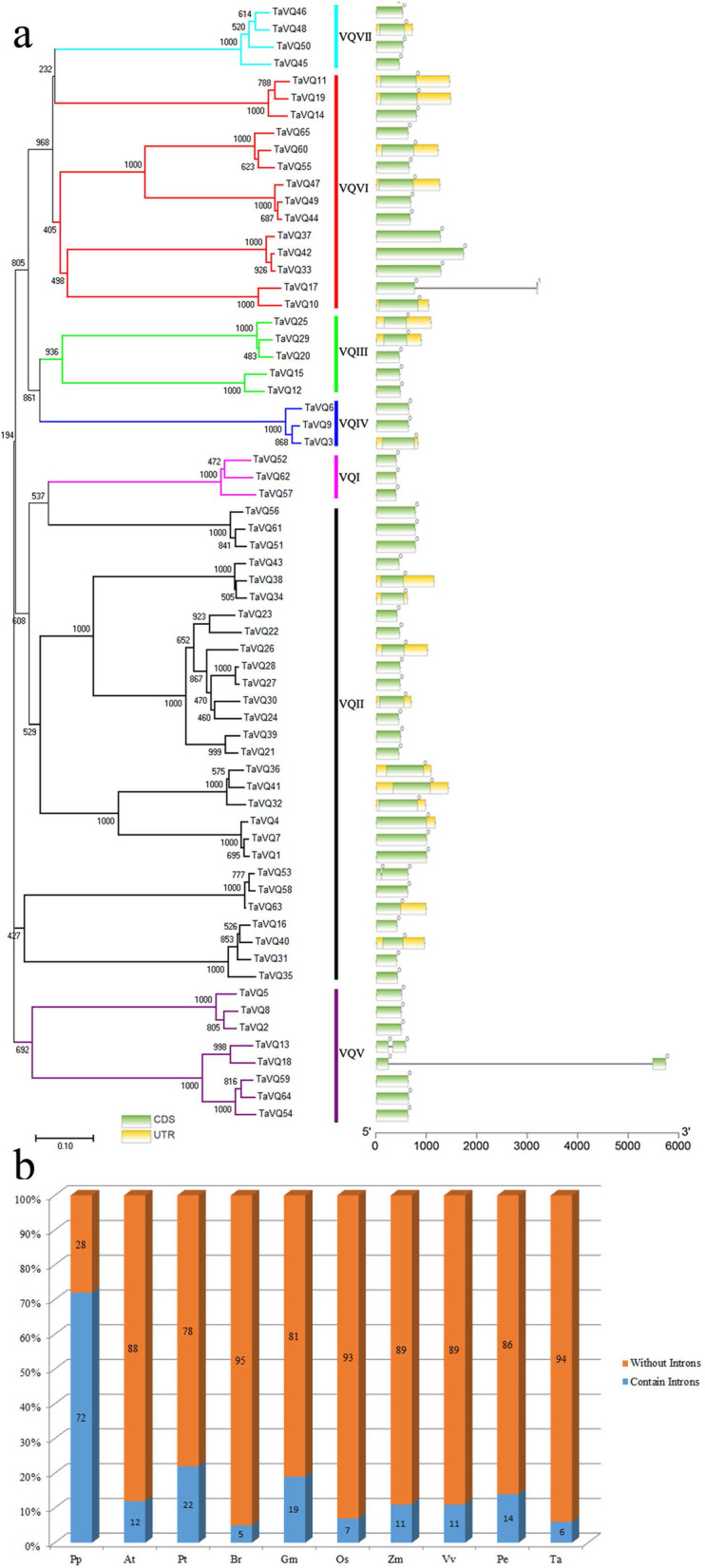
Gene structure analysis of *TaVQ* genes. **a** Phylogenetic relationships and gene structures of *TaVQ* genes. Exons, introns, and untranslated regions (UTRs) are indicated by green rectangles, gray lines, and yellow rectangles, respectively. Coloured boxes indicate the subfamily based on the phylogenetic analysis. **b** The numbers of VQ genes and the numbers of VQ genes without introns in different species. Pp: Moss, At: *Arabidopsis*, Pt: Poplar, Br: Chinese Cabbage, Gm: Soybean, Os: Rice, Zm: Maize, Vv: Grape, Pe: Moso bamboo, Ta: Wheat

We constructed a wheat *VQ* phylogenetic tree and a gene structure diagram (Fig. 2a). The structure of each *VQ* gene contained one to three parts: the untranslated region (yellow rectangle), the exon region (green rectangle), and the intron region (solid gray line). Among the 48 *TaVQ* paralogous pairs (Table 2), only two pairs (*TaVQ10/-17* and *TaVQ53/-58*) differed in intron number, having lost or gained one intron (Fig. 2A and Table S2). Further analysis revealed that 94% of the *TaVQ* genes had no introns, and only four genes (*TaVQ13/-17/-18/-53*) contained one intron. This result is consistent with previous studies in other species: 78%, 88%, 89% and 93% of the *VQ* genes in poplar, *Arabidopsis*, maize, and rice have no introns, respectively, whereas only 28% of moss *VQ* genes have no introns (Fig. 2b). Based on comparisons of many species, including angiosperms (rice, poplar, soybean, Chinese cabbage, etc.) and bryophytes (moss), we speculate that most *VQ* genes tend to lose introns during long-term evolution [6, 8, 9, 43, 44].

**Table 2:** Paralogous (Ta/Ta) and orthologous (Ta/Os and Ta/Zm) gene pairs

Paralogous	Orthologous	
TaVQ1/TaVQ4	TaVQ1/OsVQ8	TaVQ1/ZmVQ14
TaVQ1/TaVQ7	TaVQ1/OsVQ15	TaVQ1/ZmVQ59
TaVQ3/TaVQ6	TaVQ3/OsVQ16	TaVQ4/ZmVQ14
TaVQ3/TaVQ9	TaVQ4/OsVQ15	TaVQ4/ZmVQ59
TaVQ4/TaVQ7	TaVQ6/OsVQ16	TaVQ6/ZmVQ13
TaVQ6/TaVQ9	TaVQ7/OsVQ8	TaVQ7/ZmVQ14
TaVQ10/TaVQ17	TaVQ7/OsVQ15	TaVQ7/ZmVQ59
TaVQ11/TaVQ14	TaVQ9/OsVQ16	TaVQ32/ZmVQ30
TaVQ11/TaVQ19	TaVQ10/OsVQ12	TaVQ41/ZmVQ30
TaVQ12/TaVQ15	TaVQ17/OsVQ12	TaVQ53/ZmVQ35
TaVQ13/TaVQ18	TaVQ26/OsVQ26	TaVQ53/ZmVQ51
TaVQ14/TaVQ19	TaVQ32/OsVQ8	TaVQ58/ZmVQ35
TaVQ21/TaVQ39	TaVQ33/OsVQ35	TaVQ58/ZmVQ51
TaVQ22/TaVQ23	TaVQ36/OsVQ8	TaVQ63/ZmVQ35
TaVQ22/TaVQ26	TaVQ37/OsVQ35	
TaVQ22/TaVQ27	TaVQ41/OsVQ8	
TaVQ22/TaVQ28	TaVQ44/OsVQ5	
TaVQ22/TaVQ30	TaVQ47/OsVQ5	
TaVQ24/TaVQ26	TaVQ49/OsVQ5	
TaVQ24/TaVQ27	TaVQ53/OsVQ23	
TaVQ24/TaVQ28	TaVQ58/OsVQ23	
TaVQ24/TaVQ30	TaVQ63/OsVQ23	
TaVQ26/TaVQ27		
TaVQ26/TaVQ28		
TaVQ26/TaVQ30		
TaVQ27/TaVQ28		
TaVQ27/TaVQ30		
TaVQ27/TaVQ39		
TaVQ28/TaVQ30		
TaVQ28/TaVQ39		
TaVQ32/TaVQ36		
TaVQ32/TaVQ41		
TaVQ33/TaVQ37		
TaVQ33/TaVQ42		
TaVQ36/TaVQ41		
TaVQ37/TaVQ42		
TaVQ44/TaVQ47		
TaVQ44/TaVQ49		
TaVQ47/TaVQ49		
TaVQ51/TaVQ56		
TaVQ51/TaVQ61		
TaVQ53/TaVQ58		
TaVQ54/TaVQ59		
TaVQ55/TaVQ60		
TaVQ55/TaVQ65		
TaVQ56/TaVQ61		
TaVQ59/TaVQ64		
TaVQ60/TaVQ65		

**Fig. 3:**
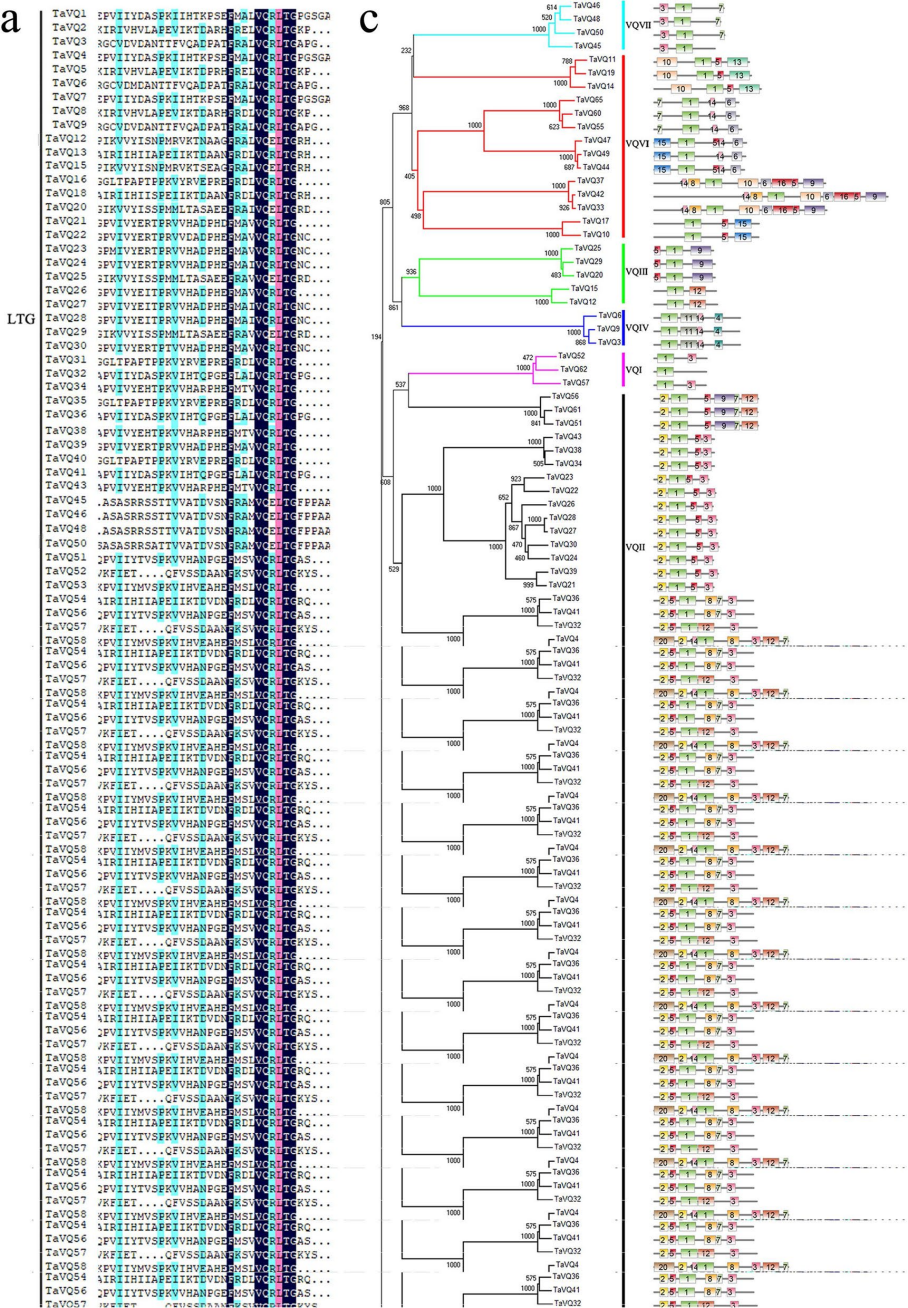
Multiple sequence alignment of TaVQ proteinsand domain type analysis in different species. **a** Multiple sequence alignment of VQ proteins in wheat. **b** VQ domain type in different species. Pp: Moss, At: *Arabidop*sis, Pt: Poplar, Br: Chinese Cabbage, Gm: Soybean, Os: Rice, Zm: Maize, Vv: Grape, Pe: Moso bamboo, Ta: Wheat. **c** Schematic representation of 20 conserved motifs in the TaVQ genes. Different colored boxes represent different motifs. Box lengths are not proportional to actual motif size

Using published information on characteristics of the VQ domain as a reference, we aligned the protein sequences of wheat and analyzed their VQ domains. The 65 TaVQ proteins all contained conserved VQ domains, but they differed slightly and could be grouped into three types: FxxxVQxLTG (52/65), FxxxVQxFTG (10/65), and FxxxVQxITG (3/65) (Fig. 3a). We further analyzed the VQ domains from multiple species (Fig. 3b) and found that the FxxxVQxLTG sequence was most prevalent and that two additional VQ domain types (LTG/FTG) were also common. There were differences in VQ domain sequence between monocots and dicots. In addition to the more common domain sequences, monocot VQ domains also included ITG, ATG, and LTA, and dicot VQ domains included LTS, LTD, YTG, LTR, and LTV (Table S3).

A total of 20 conserved motifs were identified in the *TaVQ* gene family (Table S4). All 65 TaVQ proteins shared one conserved motif (core motif 1, Motif 1) (Fig. 3c). The MEME diagram showed that wheat *VQ* genes from the same subfamily tended to share the same conserved motifs. Only 7 of the 48 *TaVQ* paralogous pairs (*TaVQ12/-15*, *TaVQ32/-36*, *TaVQ32/-41*, *TaVQ44/-49*, *TaVQ47/-49*, *TaVQ55/-60*, and *TaVQ60/-65*) differed in their motifs.

### Evolution and divergence of the *VQ* gene family in wheat, rice, and maize

In total, 48 homologous pairs were identified in wheat, 22 in wheat and rice, and 14 in wheat and maize (Table 2). The Ks (number of synonymous substitutions per synonymous site) values of the wheat paralogous pairs ranged from 0.0163 to 1.5197, indicating that duplication events occurred in this species approximately 1.2538 to 116.8985 million years ago (MYA). The Ks values of orthologous pairs from wheat and rice ranged from 0.5821 to 1.6479, indicating that duplication events occurred approximately 44.78 to 126.7631 MYA. The Ks values of orthologous pairs from wheat and maize ranged from 0.6453 to 1.0257, indicating that the duplication events occurred approximately 49.6415 to 78.8962 MYA (Table 3).

**Table 3:** Ka, Ks and Ka/Ks ratios of paralogous and orthologous pairs

Pairs	Ka	Ks	Ka/Ks	Date(MYA)
TaVQ1/TaVQ4	0.0127	0.1185	0.1070	9.1162
TaVQ1/TaVQ7	0.0070	0.0894	0.0790	6.8792
TaVQ3/TaVQ6	0.0240	0.0751	0.3200	5.7800
TaVQ3/TaVQ9	0.3823	0.2480	1.5420	19.0754
TaVQ4/TaVQ7	0.0085	0.0935	0.0900	7.1931
TaVQ6/TaVQ9	0.3781	0.2406	1.5720	18.5046
TaVQ10/TaVQ17	0.0448	0.0747	0.5990	5.7446
TaVQ11/TaVQ14	1.8069	0.9075	1.9910	69.8100
TaVQ11/TaVQ19	1.0034	0.6031	1.6640	46.3915
TaVQ12/TaVQ15	0.2352	0.1872	1.2560	14.4008
TaVQ13/TaVQ18	0.4955	0.3656	1.3550	28.1223
TaVQ14/TaVQ19	2.2662	0.9324	2.4310	71.7200
TaVQ21/TaVQ39	1.1545	0.9827	1.1750	75.5954
TaVQ22/TaVQ23	1.1107	0.9339	1.1890	71.8415
TaVQ22/TaVQ26	0.9021	0.8832	1.0210	67.9385
TaVQ22/TaVQ27	0.9769	0.8069	1.2110	62.0700
TaVQ22/TaVQ28	0.9796	0.8010	1.2230	61.6123
TaVQ22/TaVQ30	0.9757	0.8110	1.2030	62.3854
TaVQ24/TaVQ26	0.8192	0.8369	0.9790	64.3792
TaVQ24/TaVQ27	0.9598	0.8863	1.0830	68.1777
TaVQ24/TaVQ28	0.9475	0.8613	1.1000	66.2500
TaVQ24/TaVQ30	0.4472	0.4703	0.9510	36.1738
TaVQ26/TaVQ27	0.5848	0.5587	1.0470	42.9754
TaVQ26/TaVQ28	0.5786	0.5392	1.0730	41.4792
TaVQ26/TaVQ30	0.9816	0.9084	1.0810	69.8746
TaVQ27/TaVQ28	0.0059	0.0163	0.3640	1.2538
TaVQ27/TaVQ30	0.9130	0.8141	1.1210	62.6192
TaVQ27/TaVQ39	0.6100	0.4961	1.2300	38.1592
TaVQ28/TaVQ30	0.9005	0.7965	1.1310	61.2700
TaVQ28/TaVQ39	0.6039	0.4944	1.2210	38.0331
TaVQ32/TaVQ36	0.9418	0.5073	1.8560	39.0254
TaVQ32/TaVQ41	0.8366	0.6503	1.2860	50.0215
TaVQ33/TaVQ37	1.3991	1.0467	1.3370	80.5123
TaVQ33/TaVQ42	2.5055	1.2491	2.0060	96.0877
TaVQ36/TaVQ41	0.8192	0.6094	1.3440	46.8800
TaVQ37/TaVQ42	2.1547	1.5197	1.4180	116.8985
TaVQ44/TaVQ47	0.2084	0.1818	1.1470	13.9815
TaVQ44/TaVQ49	0.2361	0.1606	1.4700	12.3569
TaVQ47/TaVQ49	0.2438	0.1783	1.3670	13.7138
TaVQ51/TaVQ56	0.2983	0.3403	0.8770	26.1769
TaVQ51/TaVQ61	0.2983	0.3403	0.8770	26.1769
TaVQ53/TaVQ58	1.4092	0.5952	2.3680	45.7823
TaVQ54/TaVQ59	0.9133	0.4927	1.8540	37.8962
TaVQ55/TaVQ60	1.2444	0.5470	2.2750	42.0762
TaVQ55/TaVQ65	1.2015	0.4813	2.4960	37.0231
TaVQ56/TaVQ61	0.1112	0.1833	0.6060	14.1023
TaVQ59/TaVQ64	1.0923	0.6707	1.6290	51.5885
TaVQ60/TaVQ65	0.1719	0.1390	1.2370	10.6892
TaVQ1/OsVQ8	2.7423	0.8121	3.3770	62.4654
TaVQ1/OsVQ15	1.7626	1.1060	1.5940	85.0769
TaVQ3/OsVQ16	2.7350	0.8081	3.3840	62.1623
TaVQ4/OsVQ15	1.7960	1.0882	1.6500	83.7077
TaVQ6/OsVQ16	2.5321	0.8356	3.0300	64.2731
TaVQ7/OsVQ8	2.6174	0.8540	3.0650	65.6892
TaVQ7/OsVQ15	1.7711	1.1700	1.5140	89.9977
TaVQ9/OsVQ16	2.1032	0.7993	2.6310	61.4815
TaVQ10/OsVQ12	1.5611	0.9874	1.5810	75.9523
TaVQ17/OsVQ12	1.5725	1.0693	1.4710	82.2562
TaVQ26/OsVQ26	1.8812	1.6479	1.1420	126.7631
TaVQ32/OsVQ8	2.3258	0.8401	2.7680	64.6254
TaVQ33/OsVQ35	1.6720	0.9193	1.8190	70.7123
TaVQ36/OsVQ8	2.7247	1.0176	2.6780	78.2731
TaVQ37/OsVQ35	1.6260	0.8935	1.8200	68.7277
TaVQ41/OsVQ8	2.0860	0.9970	2.0920	76.6931
TaVQ44/OsVQ5	1.3857	0.6703	2.0670	51.5615
TaVQ47/OsVQ5	1.1333	0.6778	1.6720	52.1400
TaVQ49/OsVQ5	1.0462	0.5916	1.7680	45.5054
TaVQ53/OsVQ23	1.8754	0.6018	3.1160	46.2923
TaVQ58/OsVQ23	1.6660	0.5821	2.8620	44.7800
TaVQ63/OsVQ23	1.8612	0.7183	2.5910	55.2562
TaVQ1/ZmVQ14	1.7229	0.7294	2.3620	56.1085
TaVQ1/ZmVQ59	2.1932	0.9246	2.3720	71.1208
TaVQ4/ZmVQ14	1.8287	0.7525	2.4300	57.8877
TaVQ4/ZmVQ59	2.3077	0.9715	2.3750	74.7292
TaVQ6/ZmVQ13	1.9158	1.0257	1.8680	78.8962
TaVQ7/ZmVQ14	1.7985	0.7603	2.3660	58.4823
TaVQ7/ZmVQ59	2.3096	0.8740	2.6430	67.2292
TaVQ32/ZmVQ30	1.1674	0.8904	1.3110	68.4954
TaVQ41/ZmVQ30	2.1318	0.8361	2.5500	64.3131
TaVQ53/ZmVQ35	1.0682	0.6453	1.6550	49.6415
TaVQ53/ZmVQ51	2.1978	0.7752	2.8350	59.6277
TaVQ58/ZmVQ35	2.1378	0.8635	2.4760	66.4215
TaVQ58/ZmVQ51	1.3189	0.7664	1.7210	58.9500
TaVQ63/ZmVQ35	3.0729	0.7873	3.9030	60.5592

To investigate the role of natural selection in the evolution of the *VQ* gene family in Gramineae, we analyzed the Ka (number of non-synonymous substitutions per non-synonymous site)/Ks ratios of all homologous pairs and generated sliding window graphs (Figure S1 and Table 3). Among the 48 paralogous pairs, 11 had Ka/Ks ratios less than one, and 37 pairs had Ka/Ks ratios greater than one, indicating that wheat *VQ* genes were mainly under positive selection during the evolutionary process. The Ka/Ks ratio of all orthologous pairs was greater than one, indicating that the *VQ* gene family in wheat, rice, and maize had primarily undergone positive selection.

### Expression pattern analysis of the *TaVQ* gene family

**Fig. 4:**
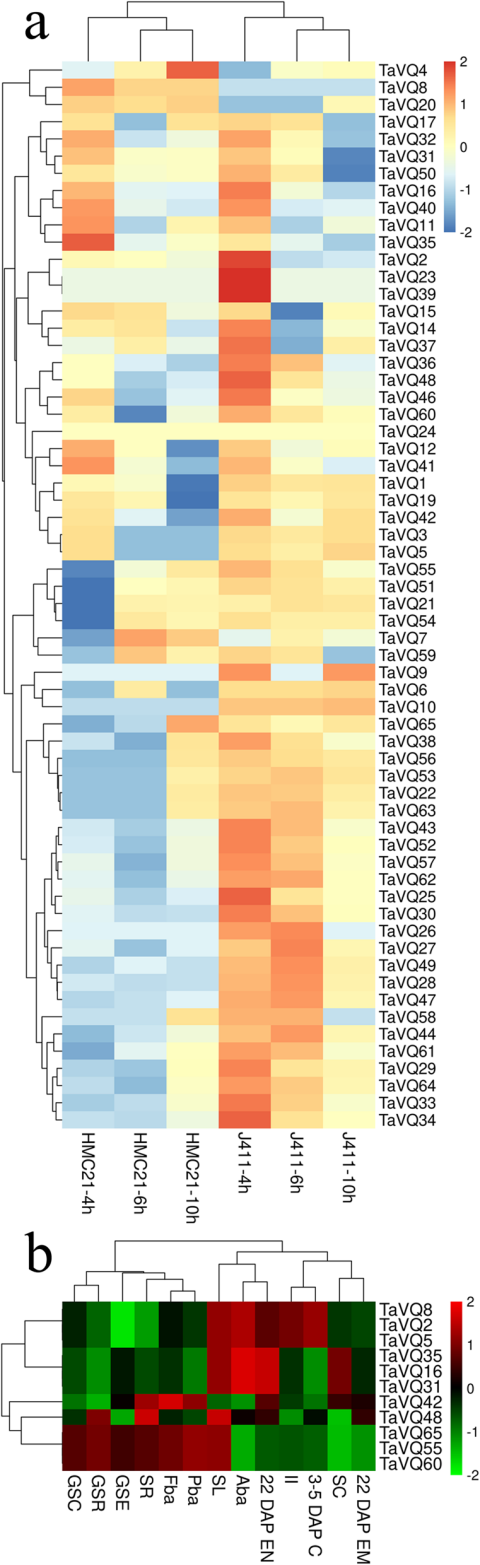
Expression profiles of *TaVQ* genes in different tissues and at different developmental stages. **a** Heatmap shows hierarchical clustering of the 62 *TaVQ* genes based on transcriptome results. **b** Heatmap shows hierarchical clustering of 11*TaVQ* genes from different tissues. Abbreviations represent specific developmental stages. GSC, germinating seed coleoptile; GSR, germinating seed root; GSE, germinating seed, embryo; SR, seedling root; SC, seedling crown; SL, seedling leaf; II, immature inflorescence; Fba, floral bracts before anthesis; Pba, pistil before anthesis; Aba, anthers before anthesis; 3–5 DAP C, 3–5 DAP caryopsis; 22 DAP EM, 22 DAP embryo; 22 DAP EN, 22 DAP endosperm

Transcriptome data (FPKM values) were obtained for all *TaVQ* genes with the exception of *TaVQ13/-18/-45* (Fig. 4a and Table S5). The expression patterns of VQ genes differed among varieties and within time periods in the same variety. Most *TaVQ* genes were highly expressed in J411, especially at 4 h after seed imbibition, and only four genes (*TaVQ4/-7/-8/-20*) were expressed at a low level. It is worth noting that *TaVQ8* and *TaVQ20* were both highly expressed in HMC21 and expressed at a low level in J411. We further analyzed the expression of 48 paralogous gene pairs. Only one pair showed a similar expression pattern, whereas the rest were differentially expressed among varieties and within different time periods of the same variety.

Microarray data were obtained for 11 *TaVQ* genes to further investigate wheat *VQ* gene expression (Fig. 4b and Table S6). *TaVQ16*, *TaVQ31*, and *TaVQ35* were highly expressed in Aba and at 22 DAP EM (22 days after planting—embryo), but they showed little expression elsewhere. Further analysis of paralogous pairs showed that three pairs (*TaVQ55/-60*, *TaVQ55/-65*, and *TaVQ60/-65*) had similar expression patterns in different tissues.

### Promoter analysis and gene ontology annotation of the *TaVQ* gene family

**Fig. 5:**
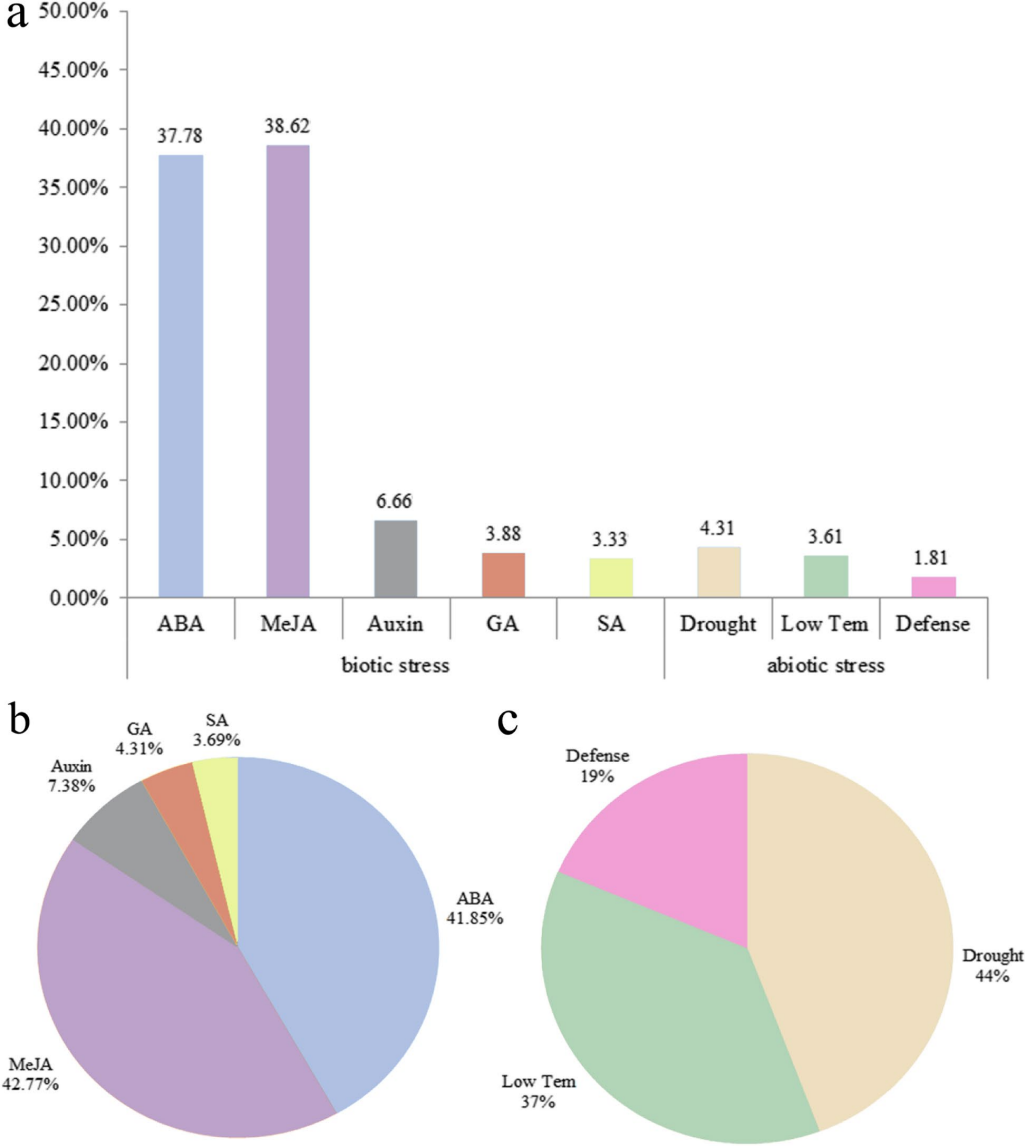
*Cis*-acting element analysis of the promoter regions of *TaVQ* genes. Based on functional annotation data, *cis*-acting elements were classified into two major classes: phytohormone responsive elements (i.e. those responsive to ABA, auxin, GA, MeJA, and/or SA) and abiotic stress responsive elements (e.g. those involved in plant defense, drought stress response, and/or low temperature stress response). **a** Percentage of total *cis*-acting elements in the promoter region of the *TaVQ* gene. **b** and **c** The percentage of *cis*-acting elements in different categories

Two categories of response element were analyzed in the promoter regions of the *TaVQ* genes (Fig. 5a and Table S7). The first category included elements associated with biotic stress, such as ABRE, CGTCA motif, TGACG motif, TGA element, AuxRR core, TCA element, GARE motif, and P-box. The second category included elements associated with abiotic stress, such as MBS, LTR, and TC-rich repeats. The most common biotic stress response elements in the *TaVQ* promoters were associated with methyl jasmonate (CGTCA motif and TGACG motif) (42.77%) and ABA (ABRE) (41.85%) (Fig. 5b). The drought-associated MBS element (4.31%) was the most common abiotic stress response element (Fig. 5c).

**Fig. 6:**
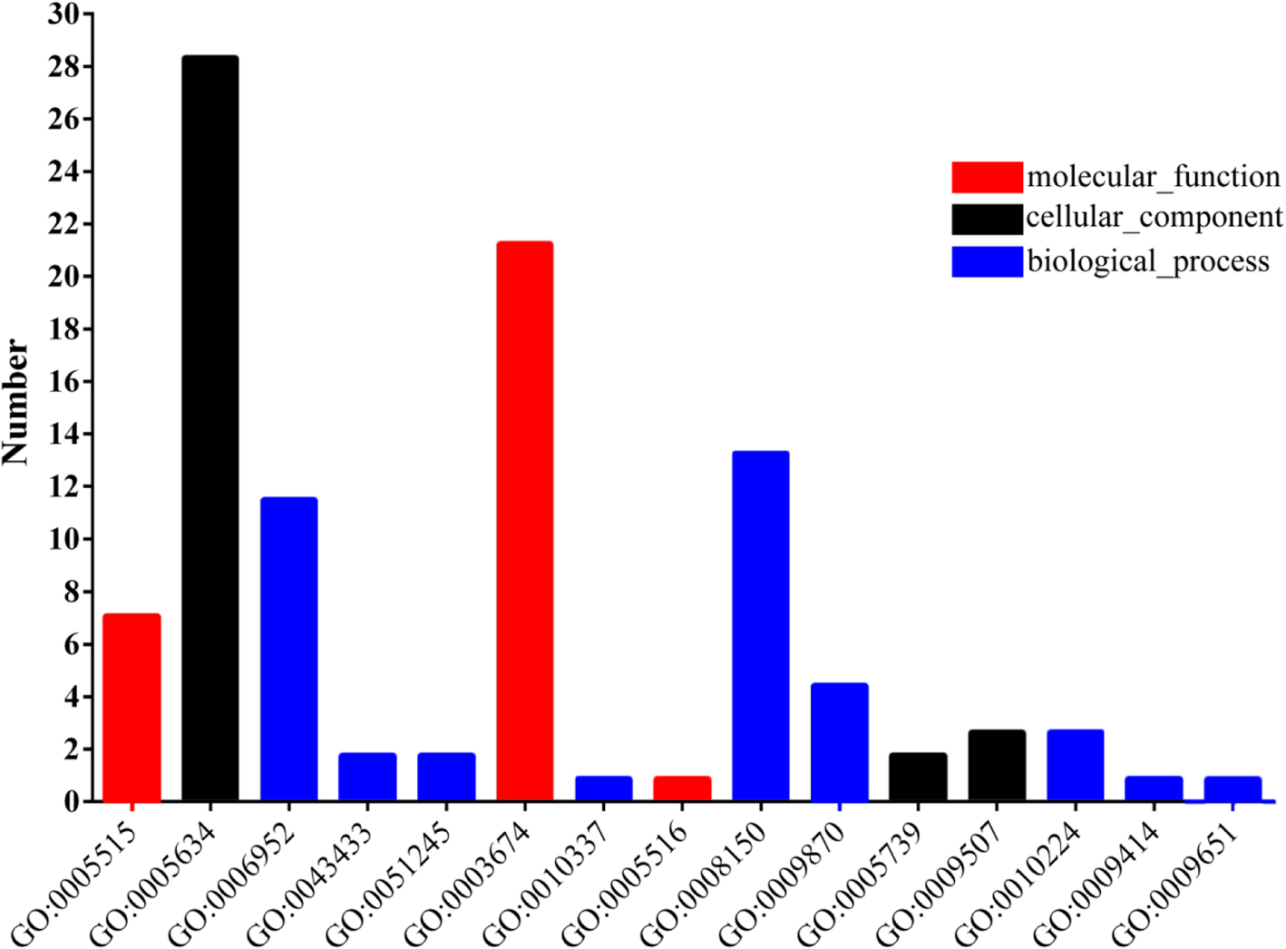
Gene Ontology (GO) annotations of TaVQ proteins. Red represents molecular_function, blue represents biological_process, black represents cellular_component

The 65 *TaVQ* genes were annotated with 15 GO terms (Fig. 6 and Table S8): three, three, and nine terms in the molecular function, cellular component, and biological process categories, respectively. Among these terms, GO:0,005,634 (cellular component), GO:0,003,674 (molecular function), and GO:0,008,150 (biological process) were most common and were assigned to 28, 21, and 13 genes, respectively.

### Chromosome locations and subcellular localization predictions for the *TaVQ* gene family

The *TaVQ* genes were unevenly distributed on wheat chromosomes 1–7, and no *TaVQ* genes were present on chromosomes 1B and 1D (Figure S2). One gene was located on chromosome 1A, five were located on chromosomes 5B, 7A, 7B, and 7D, and two to four were located on each of the other chromosomes. We defined a single gene cluster as a chromosomal region of less than 200 kb that contained two or more *TaVQ* genes [45]. Two gene clusters containing six genes were identified on chromosomes 4A and 4B (Figure S2).

Subcellular localization prediction indicated that the TaVQ proteins were present in three locations. Most were predicted to be located in the periplasmic region (47, 72.3%), some in the extracellular region (15, 23.1%), and the rest in the cytoplasm (3, 4.6%) (Table S9).

### Responses of *TaVQ* genes to water imbibition

**Fig. 7:**
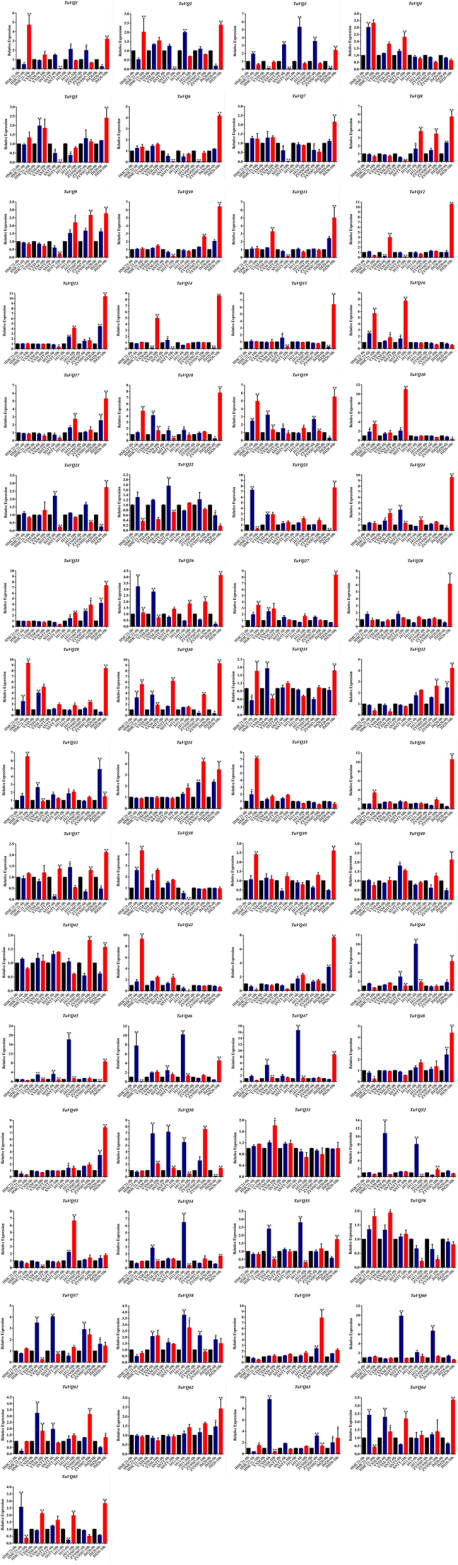
Relative expression (mean ± SE) of 65*TaVQ* genes during seed imbibition in six wheat varieties

We investigated the responses of 65 *TaVQ* genes (Table 1 and Table S10) in six wheat varieties with different seed dormancy and germination phenotypes after water imbibition for 0, 6, and 10 h. Seeds from three highly dormant varieties (HMC21, YXM, and SNTT) showed no seed germination, whereas partial seeds from three low-dormancy varieties (J411, ZY9507, and ZM895) germinated after 10 h of imbibition with an average germination index (GI) of 0.33, 0.31, and 0.41, respectively (Table S11). We found that the *TaVQ* genes were differentially expressed in the six wheat varieties. The expression levels of 13 genes (*TaVQ8/-9/-13/-17/-25/-32/-34/-43/-48/-49/-53/-59/-62*) were higher in the low-dormancy varieties than in the high-dormancy varieties. Eight genes showed the opposite expression trend (*TaVQ4/-16/-20/-35/-38/-42/-51/-56*) (Fig. 7).

## Discussion

The plant-specific VQ proteins initially attracted attention due to their interactions with WRKY transcription factors [15]. Additional in-depth studies showed that the VQ gene family not only participated in responses to biotic and abiotic stress, but was also involved in the regulation of plant growth and development [5–7, 13–17]. Genome-wide surveys of VQ proteins have now been performed in a number of species, although functional research has remained focused on *Arabidopsis*. *VQ* genes have not previously been characterized in wheat, and we therefore performed basic bioinformatics analyses to better understand the *VQ* gene family in wheat.

We identified 65 *VQ* genes from wheat and classified them into seven subfamilies. The *VQ* genes of five species (wheat, rice, maize, poplar, and *Arabidopsis*) were distributed in each subfamily, but the number of subfamily members differed among species, indicating that the *VQ* genes have developed in multiple directions over the course of evolution. The *VQ* genes of monocots (rice, maize, and wheat) were interspersed and clustered together, whereas the *VQ* genes of *Arabidopsis* and poplar were clustered into separate clades, indicating that proteins encoded by wheat *VQ* genes were highly similar to those of rice and maize [8, 9]. These results highlight the evolutionary conservation of the *VQ* gene family.

Phylogenetic trees represent the genetic relationships among gene families from different species and reflect the similarity of protein-coding genes. From structural analysis of the *VQ* gene family, we found that most *VQ* genes were intron-free [6, 8–11, 43, 44]. Based on comparisons of several species, we speculate that this gene family tends to lose introns during evolution. Amino acid sequence alignment and motif analysis indicated that the sequences of most VQ domains from different species were similar, although a small number of variants existed. In general, members of the same subfamily had similar types and numbers of conserved motifs, but there were also cases in which members of the same family had different types and numbers of conserved motifs. In addition, VQ had mutated to VH in the VQ domain of several Gramineae species [5, 6]. Taken together, these results indicate that the *VQ* gene family is highly conserved and diverse, reflecting the functional diversity of the gene family members.

With the development of next generation sequencing technology, the genomes of *Arabidopsis*, rice, maize, and wheat have recently been sequenced [2, 3, 8–10]. Their genome sizes are 164 Mb, 389 Mb, 2500 Mb, and 17 Gb, respectively. Based on genome size and chromosome number, the number of *VQ* genes among the four species is expected to be the highest in wheat, followed by maize, rice, and *Arabidopsis*. The numbers of maize, rice, and *Arabidopsis VQ* genes are 61, 40, and 34, and the number of wheat *VQ* genes in this study was 65, consistent with predictions based on genome size. By calculating the Ks value of homologous pairs to estimate the time of duplication events, the time range for whole genome duplication events in wheat was approximately 1.2538 to 116.8985 MYA. The Ka/Ks ratio of most paralogous pairs (37, 77%) was greater than one, indicating that the *TaVQ* gene family had undergone positive selection. A sliding window graph demonstrated that the Ka/Ks ratio of homologous pairs differed among different coding segments: some had Ka/Ks ratios greater than one, and some had Ka/Ks ratios less than one, indicating that the homologous pairs had undergone different evolutionary selection pressures. These results show that natural selection has played an important role in the evolution and differentiation of the *VQ* gene family.

*TaVQ55/-60/-65* were highly expressed in GSC, GSR, GSE, SR, SL, Fba, and Pba; *TaVQ2/-5/-8* were highly expressed in SL, Aba, 3–5 DAP C (22 days after planting—caryopsis), and 22 DAP EM; and *TaVQ16/-31/-35* were highly expressed in SL, Aba, and 22 DAP EM. These results indicate that the VQ gene family is active during multiple plant growth and developmental stages. Previous studies on *Arabidopsis* have shown that *IKU1* (*AtVQ14*, *At2g35230*) regulates endosperm development and seed size [20]. In this study, *TaVQ48*, which belongs to the same subfamily as *AtVQ14/-29*, was also expressed in floral bracts before anthesis, in 22 DAP EN, and in 22 DAP EM. In addition, *TaVQ48* was strongly expressed in germinating seeds, roots, seedling roots, and seedling leaves. These results will guide further exploration of the functions of the *TaVQ* gene family.

GO annotations of 62 *TaVQ* genes were extracted from transcriptome data. The most common GO terms were from the biological process category (43, 38.1%), especially GO:0,006,952 (13 genes) and GO:0,008,150 (15 genes). GO:0,006,952 is related to defense response, and combined with promoter analysis, we found that 5 *TaVQ* genes (*TaVQ1/-2/-32/-41/-51*) had this function in both analyses. GO:0,010,337 is related to the regulation of salicylic acid (SA) metabolism, but only *TaVQ14* was assigned this annotation. *TaVQ14* also had an SA *cis*-acting element in the promoter analysis. These results indicate that gene structure determines function, and the diversity of structure reflects the diversity of function.

In the present study, we measured the expression of the 65 *TaVQ* genes during seed imbibition of six wheat varieties (HMC21, YXM, SNTT, J411, ZY9507, and ZM895). The expression of thirteen *TaVQ* genes (*TaVQ8/-9/-13/-17/-25/-32/-34/-43/-48/-49/-53/-59/-62*) was consistently higher in low-dormancy varieties than in high-dormancy varieties. By contrast, the expression levels of 8 *TaVQ* genes (*TaVQ4/-16/-20/-35/-38/-42/-51/-56*) were consistently higher in high-dormancy varieties. These 21 *TaVQ* genes may therefore participate in the regulation of seed dormancy and germination. According to phylogenetic analysis, three of these 21 genes (*TaVQ8*, *TaVQ13*, and *TaVQ59*) are members of the VQV subfamily. Interestingly, *Arabidopsis AtVQ18* and *AtVQ26* involved in seed germination also belong to the VQV subfamily. These results suggest that *TaVQ8/-13/-59* may have similar functions in the regulation of seed dormancy and germination, a hypothesis that requires future validation.

## Conclusions

We investigated the phylogeny and diversification of *VQ* genes in wheat by multiple methods, including phylogenetic tree construction and characterization of gene structures, conserved domains, chromosome positions, expression patterns, and promoter elements. In addition, we measured the expression levels of *TaVQ* genes in wheat varieties with contrasting seed dormancy and germination phenotypes by qRT-PCR to identify genes that were potentially involved in seed dormancy and germination. Sixty-five TaVQ proteins were identified for the first time in common wheat, and qRT-PCR data showed that 21 were potentially involved in seed dormancy and germination. These findings provide valuable information for further cloning and functional analysis of *TaVQ* genes, as well as useful candidate genes for improvement of PHS resistance in wheat.

## Methods

### Plant materials

We measured *TaVQ* gene expression in six wheat varieties with extreme dormancy levels [46]: J411 (Jing 411, average germination index [GI] = 0.89, average germination rate [GR] = 98.00%), HMC21 (Hongmangchun 21, average GI = 0.04, average GR = 10.00%), SNTT (Suiningtuotuo, average GI = 0.06, average GR = 16.00%), ZM895 (Zhongmai 895, average GI = 0.81, average GR = 96.00%), ZY9507 (Zhongyou 9507, average GI = 0.90, average GR = 98.00%), and YXM (Yangxiaomai, average GI = 0.03, average GR = 9.00%) (Tables S11 and S12). J411 and HMC21 were provided by Shihe Xiao from the Chinese Academy of Agricultural Sciences, and ZM895, ZY9507, YXM, and SNTT were provided by Xianchun Xia from the Chinese Academy of Agricultural Sciences.

### Germination index and germination rate assays

Freshly harvested seeds were used to measure the GI as described in our previous study [46]. Fifty seeds from each genotype were placed in Φ 90 Petri dishes on filter paper with 9 ml distilled water, then grown in a 20 °C greenhouse with a 14 h day/10 h night photoperiod at 80% humidity. The number of germinated seeds in each culture dish was counted at the same time every day, and germinated seeds were removed. The GI value was calculated after 3 days as GI = ([3 × n1] + [2 × n2] + [3 × n1])/3 × N. The GR was also calculated after 3 days of seed imbibition as GR = [(n1 + n2 + n3)/N] × 100%. In these equations, n1, n2, and n3 are the numbers of seeds germinated on the first, second, and third days, and N is the total number of seeds. Each genotype was replicated three times, and germination was defined as visible rupture of the pericarp and testa.

### Identification of wheat *VQ* genes

To determine the number of *VQ* genes in common wheat, we used sequences obtained from the Ensembl database to build a local wheat database [46]. The VQ domain hidden Markov model (PF05678) was used to identify candidate genes by BLAST in the established local wheat database. To ensure the accuracy of the results, all candidate genes were inspected, repetitive sequences were removed, and Pfam, SMART, and NCBI online tools were used to verify the existence of the conserved VQ domain in all candidate genes [46, 47]. The ExPASy online tool was used to predict the isoelectric point (PI), protein molecular weight (MW), open reading frame (ORF), and other attributes of the VQ proteins.

### Phylogenetic tree and multiple sequence alignment

FASTA sequence files were opened in ClustalX2.11 software [48–51] and used to generate a multiple sequence alignment from which a phylogenetic tree was constructed using the neighbor-joining method with 1000 bootstrap replicates in MEGA7.0 [43, 52–54]. The same method was used to build a composite phylogenetic tree of VQ protein sequences from maize, rice, poplar, *Arabidopsis*, and wheat.

### Intron/exon structure and conserved motif analysis

The distribution and structure of exons and introns were determined by uploading CDS and genomic sequences to the Gene Structure Display Server (http://gsds.cbi.pku.edu.cn/) for plotting and analysis [7, 54, 55].

To predict structural differences among the TaVQ proteins, all candidate protein sequences were uploaded to the MEME online tool (http://memesuite.org/tools/meme) for conserved motif analysis using standard operating parameters [54, 56].

### Identification of homologous pairs and calculation of Ka/Ks values

Using previously reported methods for the identification of homologous gene pairs (paralogs and orthologs), the nucleotide sequences of *VQ* genes from wheat and other species were compared using BLASTN [57, 58].

Wheat homologous gene pairs were compared and aligned in ClustalX 2.11, and the aligned sequences were analyzed in MEGA7.0 [59]. The results were uploaded to DnaSP v5.10.1 [60] to calculate the values of Ka (non-synonymous nucleotide mutation rate) and Ks (synonymous nucleotide mutation rate) for all homologous pairs. The formula T = (Ks/2λ) × 10^−6^ was used to estimate the approximate dates of divergence events. To further analyze Ka/Ks values, we used GraphPad Prism 5 software to generate a sliding window graph [7, 61]. A Ka/Ks ratio less than 1 indicates that a DNA mutation is harmful and under purifying selection, whereas a Ka/Ks ratio greater than 1 indicates that a DNA mutation is beneficial and under positive selection. A Ka/Ks ratio of 1 indicates neutral selection [62].

### Chromosome location and gene ontology annotation

The chromosome locations of *TaVQ* genes were downloaded from the Ensembl database, and chromosome maps were built using MapGene2Chromosome v2.0 [63]. Gene ontology (GO) annotations in the biological process, cellular component, and molecular function categories were assigned based on our transcriptome data (http://amigo.geneontology.org/amigo) [54].

### Promoter analysis and subcellular location prediction

The 1500-bp sequence upstream of the transcription start site of each *VQ* gene was downloaded from the Ensembl website, and *cis*-acting elements in the promoter region were identified using the PlantCARE online tool [64]. WOLF was used to predict the subcellular localization of the TaVQ proteins [65].

### Tissue expression pattern analysis

We collected three replicate seed tissue samples from HMC21 and J411 at 4, 6, and 10 h after seed imbibition for transcriptome sequencing. In addition, we obtained microarray data for 13 different tissues (three biological replicates each) from the Gene Expression Omnibus database (accession number GSE12508) [66, 67]. Mapper Plus was used to generate an expression heat map [45, 68].

### RNA extraction and RT-qPCR analysis

Total RNA was extracted from seeds using the TaKaRa MiniBEST Universal RNA Extraction Kit. Primer Premier 5.0 was used to design 65 *TaVQ* gene-specific primers (Table S10), and TaActin was used as the reference gene [69]. The total PCR volume was 10 μl. The reaction process was 94 °C denaturation for 30 s, followed by 40–45 cycles of 94 °C for 5 s, 50–60 °C for 15 s, and 72 °C for 10 s. We performed three biological replicates for each sample. Finally, we processed the data and created the corresponding figure in GraphPad version 5 [70].

## Supplementary Information

**Additional file 1:** **Figure S1.** Sliding window plots of the *VQ* genes. **Figure S2.** Chromosomal locations of *TaVQ* genes. Chromosome numbers are indicated above each bar. **Table S1** Detailed information about the *ZmVQ, OsVQ*, *PtVQ* and *AtVQ* genes. **Table S2.** Numbers of *VQ* genes and *VQ* genes without introns in different species. **Table S3. **VQ domain types in different species. **Table S4**. Information on 20 conserved motifs of the TaVQ protein family. **Table S5.** Transcriptome data for VQ genes. **Table S6.** Microarray data for VQ genes. **Table S7.** Promoter analysis of the TaVQ protein family. **Table S8.** Gene ontology (GO) annotations of TaVQ proteins. **Table** **S9.** Subcellular localization of *TaVQ*s predicted by WOLF PSORT. **Table S10.** qRT-PCR primers for *TaVQ* genes. **Table** **S11.** Data of seed germination index (GI) of six wheat varieties. **Table** **S12.** Data of seed germination rate (GR) of six wheat varieties.

## Data Availability

The datasets generated and analysed during the current study are available in the corresponding author on reasonable request. The genome sequences of wheat, maize, rice and *Arabidopsis* were downloaded from the Ensembl database (http://plants.ensembl.org/index.html), PlantTFDB (http://planttfdb.gao-lab.org/), Rice Genome Annotation Project database (http://rice.plantbiology.msu.edu/analyses_search_locus.shtml) and Arabidopsis Information Resource (http://www.arabidopsis.org).

## Abbreviations

VQ: Valine–glutamine
qRT-PCR: Quantitative real-time PCR
Ks: Number of synonymous substitutions per synonymous site
Ka: Number of non-synonymous substitutions per non-synonymous site
CDS: Coding sequence
bp: Base pair
aa: Amino acids
MW: Molecular weight
Da: Dalton
PHS: Pre-harvest sprouting
GI: Germination index
pI: Isoelectric point
GO: Gene ontology
GSC: Germinating seed coleoptile
GSR: Germinating seed root
GSE: Germinating seed, embryo
SR: Seedling root
SC: Seedling crown
SL: Seedling leaf
II: Immature inflorescence
Fba: Floral bracts before anthesis
Pba: Pistil before anthesis
Aba: Anthers before anthesis
3–5 DAP C: 3–5 Days after planting caryopsis
22 DAP EM: 22 Days after planting embryo
22 DAP EN: 22 Days after planting endosperm
