# Supplementary material for: Characterization of the wheat VQ protein family and expression of candidate genes associated with seed dormancy and germination
Source: BMC Plant Biol. 2022 Mar 15;22:119. doi: 10.1186/s12870-022-03430-1 (PMC8925178; doi:10.1186/s12870-022-03430-1)
Supplement: Supplementary file 1 — Additional file 1: Figure S1. Sliding window plots of the VQ genes. Figure S2. Chromosomal locations of TaVQ genes. Chromosome numbers are indicated above each bar. Table S1 Detailed information about the ZmVQ, OsVQ, PtVQ and AtVQ genes. Table S2. Numbers of VQ genes and VQ genes without introns in different species. Table S3. VQ domain types in different species. Table S4. Information on 20 conserved motifs of the TaVQ protein family. Table S5. Transcriptome data for VQ genes. Table S6. Microarray data for VQ genes. Table S7. Promoter analysis of the TaVQ protein family. Table S8. Gene ontology (GO) annotations of TaVQ proteins. Table S9. Subcellular localization of TaVQs predicted by WOLF PSORT. Table S10. qRT-PCR primers for TaVQ genes. Table S11. Data of seed germination index (GI) of six wheat varieties. Table S12. Data of seed germination rate (GR) of six wheat varieties. [file 12870_2022_3430_MOESM1_ESM.docx]

**Supporting Information files**

**
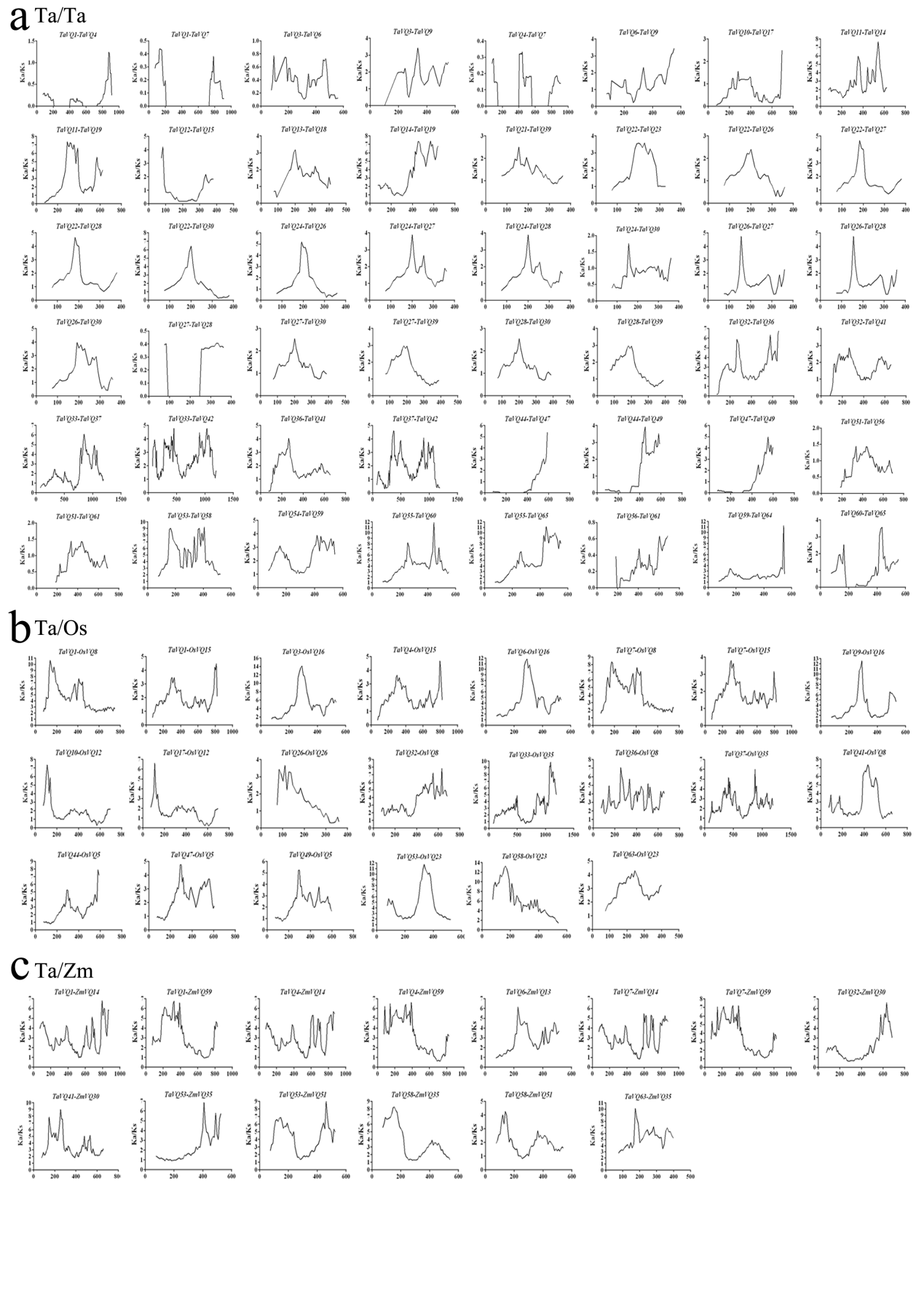

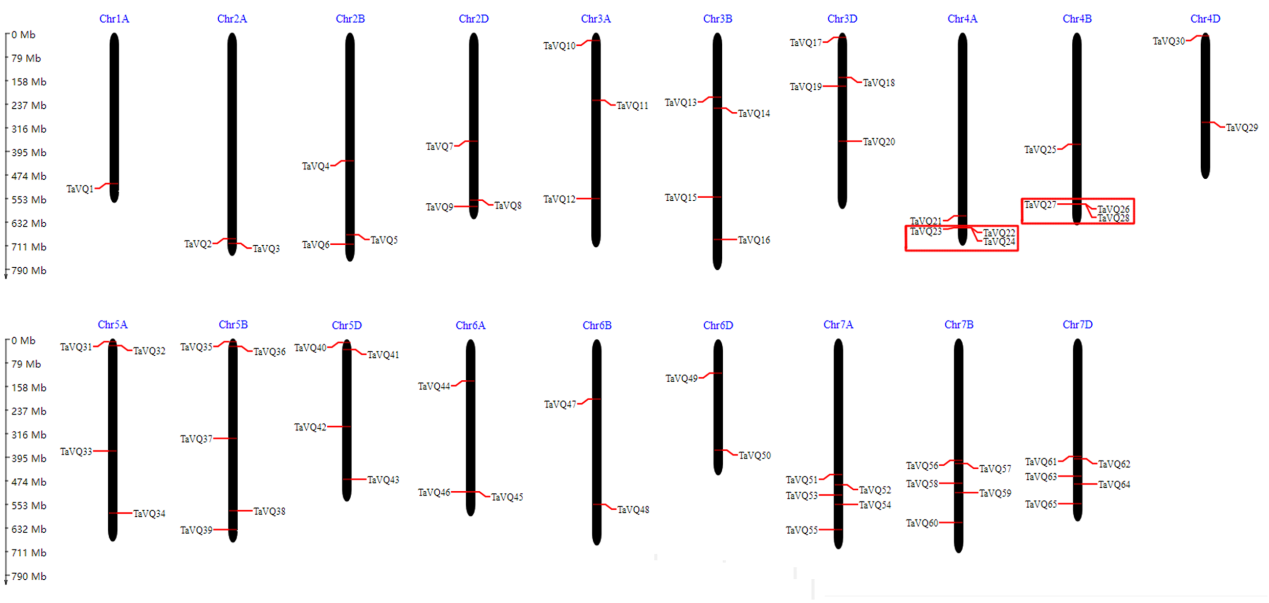
**

Figure S1. Sliding window plots of the *VQ* genes.

(a) Sliding window plots of the *VQ* genes in wheat.

(b) Sliding window plots of the *VQ* genes in wheat and rice.

(c) Sliding window plots of the *VQ* genes in wheat and maize.

Figure S2. Chromosomal locations of *TaVQ* genes. Chromosome numbers are indicated above each bar.

Table S1 Detailed information about the *ZmVQ, OsVQ*, *PtVQ* and *AtVQ* genes.

| Name | Gene ID | Name | Gene ID | Name | Gene ID | Name | Gene ID |
| --- | --- | --- | --- | --- | --- | --- | --- |
| ZmVQ1 | GRMZM2G417835 | OsVQ1 | LOC_Os01g17050 | AtVQ1 | AT1G17147 | PtVQ1 | Potri.001G029700 |
| ZmVQ2 | GRMZM2G420357 | OsVQ2 | LOC_Os01g46440 | AtVQ2 | AT1G21320 | PtVQ2 | Potri.001G130300 |
| ZmVQ3 | GRMZM2G318652 | OsVQ3 | LOC_Os01g54400 | AtVQ3 | AT1G21326 | PtVQ3 | Potri.001G142400 |
| ZmVQ4 | GRMZM2G128644 | OsVQ4 | LOC_Os01g59410 | AtVQ4 | AT1G28280 | PtVQ4 | Potri.001G158800 |
| ZmVQ5 | GRMZM2G174650 | OsVQ5 | LOC_Os02g07690 | AtVQ5 | AT1G32585 | PtVQ5 | Potri.001G230800 |
| ZmVQ6 | GRMZM2G158976 | OsVQ6 | LOC_Os02g15280 | AtVQ6 | At1G32610 | PtVQ6 | Potri.001G378300 |
| ZmVQ7 | GRMZM2G421934 | OsVQ7 | LOC_Os02g15290 | AtVQ7 | AT1G35830 | PtVQ7 | Potri.001G399100 |
| ZmVQ8 | GRMZM2G420630 | OsVQ8 | LOC_Os02g33600 | AtVQ8 | AT1G68450 | PtVQ8 | Potri.002G070600 |
| ZmVQ9 | GRMZM2G059064 | OsVQ9 | LOC_Os02g51740 | AtVQ9 | AT1G78310 | PtVQ9 | Potri.002G094900 |
| ZmVQ10 | GRMZM2G118172 | OsVQ10 | LOC_Os03g20330 | AtVQ10 | AT1G78410 | PtVQ10 | Potri.002G099900 |
| ZmVQ11 | GRMZM2G174210 | OsVQ11 | LOC_Os03g20440 | AtVQ11 | AT1G80450 | PtVQ11 | Potri.003G075800 |
| ZmVQ12 | AC206638.3_FG007 | OsVQ12 | LOC_Os03g26990 | AtVQ12 | AT2G22880 | PtVQ12 | Potri.003G091800 |
| ZmVQ13 | GRMZM2G023921 | OsVQ13 | LOC_Os03g47280 | AtVQ13 | AT2G33780 | PtVQ13 | Potri.003G194700 |
| ZmVQ14 | GRMZM2G369742 | OsVQ14 | LOC_Os03g57520 | AtVQ14 | AT2G35230 | PtVQ14 | Potri.004G044800 |
| ZmVQ15 | GRMZM2G147443 | OsVQ15 | LOC_Os04g34050 | AtVQ15 | AT2G41010 | PtVQ15 | Potri.004G071500 |
| ZmVQ16 | GRMZM2G101409 | OsVQ16 | LOC_Os04g55240 | AtVQ16 | AT2G41180 | PtVQ16 | Potri.004G134200 |
| ZmVQ17 | GRMZM2G354123 | OsVQ17 | LOC_Os04g57030 | AtVQ17 | AT2G42140 | PtVQ17 | Potri.005G057800 |
| ZmVQ18 | GRMZM2G055404 | OsVQ18 | LOC_Os05g12090 | AtVQ18 | AT2G44340 | PtVQ18 | Potri.005G076600 |
| ZmVQ19 | GRMZM2G378442 | OsVQ19 | LOC_Os05g32460 | AtVQ19 | AT3G15300 | PtVQ19 | Potri.005G162300 |
| ZmVQ20 | GRMZM2G378442 | OsVQ20 | LOC_Os05g41250 | AtVQ20 | AT3G18360 | PtVQ20 | Potri.005G166100 |
| ZmVQ21 | AC194056.3_FG008 | OsVQ21 | LOC_Os05g44270 | AtVQ21 | AT3G18690 | PtVQ21 | Potri.005G189300 |
| ZmVQ22 | GRMZM2G066599 | OsVQ22 | LOC_Os06g33970 | AtVQ22 | AT3G22160 | PtVQ22 | Potri.005G204000 |
| ZmVQ23 | GRMZM2G322950 | OsVQ23 | LOC_Os06g40090 | AtVQ23 | AT3G56710 | PtVQ23 | Potri.006G006300 |
| ZmVQ24 | GRMZM2G153597 | OsVQ24 | LOC_Os06g41450 | AtVQ24 | AT3G56880 | PtVQ24 | Potri.006G032300 |
| ZmVQ25 | GRMZM2G010333 | OsVQ25 | LOC_Os06g45570 | AtVQ25 | AT3G58000 | PtVQ25 | Potri.006G038900 |
| ZmVQ26 | GRMZM2G124290 | OsVQ26 | LOC_Os07g06750 | AtVQ26 | AT3G60090 | PtVQ26 | Potri.006G192100 |
| ZmVQ27 | GRMZM2G129140 | OsVQ27 | LOC_Os07g06760 | AtVQ27 | AT4G15120 | PtVQ27 | Potri.006G199300 |
| ZmVQ28 | GRMZM2G325208 | OsVQ28 | LOC_Os07g06790 | AtVQ28 | AT4G20000 | PtVQ28 | Potri.006G266600 |
| ZmVQ29 | AC207043.3_FG002 | OsVQ29 | LOC_Os07g43140 | AtVQ29 | AT4G37710 | PtVQ29 | Potri.006G266700 |
| ZmVQ30 | GRMZM2G346837 | OsVQ30 | LOC_Os07g48710 | AtVQ30 | AT4G39720 | PtVQ30 | Potri.007G006200 |
| ZmVQ31 | GRMZM2G061941 | OsVQ31 | LOC_Os07g48800 | AtVQ31 | AT5G08480 | PtVQ31 | Potri.007G091600 |
| ZmVQ32 | GRMZM2G003669 | OsVQ32 | LOC_Os08g01260 | AtVQ32 | AT5G46780 | PtVQ32 | Potri.007G110100 |
| ZmVQ33 | GRMZM2G420715 | OsVQ33 | LOC_Os08g31660 | AtVQ33 | AT5G53830 | PtVQ33 | Potri.009G024200 |
| ZmVQ34 | GRMZM2G082118 | OsVQ34 | LOC_Os09g20020 | AtVQ34 | AT5G65170 | PtVQ34 | Potri.010G123700 |
| ZmVQ35 | GRMZM2G099691 | OsVQ35 | LOC_Os09g20460 |  |  | PtVQ35 | Potri.011G053700 |
| ZmVQ36 | GRMZM2G174558 | OsVQ36 | LOC_Os10g01240 |  |  | PtVQ36 | Potri.011G084800 |
| ZmVQ37 | GRMZM5G814101 | OsVQ37 | LOC_Os11g03660 |  |  | PtVQ37 | Potri.011G095900 |
| ZmVQ38 | GRMZM2G355499 | OsVQ38 | LOC_Os11g12790 |  |  | PtVQ38 | Potri.011G118200 |
| ZmVQ39 | GRMZM2G083285 | OsVQ39 | LOC_Os12g03420 |  |  | PtVQ39 | Potri.012G055900 |
| ZmVQ40 | GRMZM2G126413 | OsVQ40 | LOC_Os03g09045 |  |  | PtVQ40 | Potri.013G043800 |
| ZmVQ41 | GRMZM2G316033 |  |  |  |  | PtVQ41 | Potri.014G006400 |
| ZmVQ42 | GRMZM2G151909 |  |  |  |  | PtVQ42 | Potri.014G008600 |
| ZmVQ43 | GRMZM2G036980 |  |  |  |  | PtVQ43 | Potri.014G141600 |
| ZmVQ44 | GRMZM2G180668 |  |  |  |  | PtVQ44 | Potri.015G046600 |
| ZmVQ45 | AC203294.3_FG012 |  |  |  |  | PtVQ45 | Potri.016G017900 |
| ZmVQ46 | GRMZM5G800535 |  |  |  |  | PtVQ46 | Potri.016G029600 |
| ZmVQ47 | GRMZM2G374336 |  |  |  |  | PtVQ47 | Potri.016G036600 |
| ZmVQ48 | GRMZM5G849527 |  |  |  |  | PtVQ48 | Potri.016G046000 |
| ZmVQ49 | GRMZM5G864059 |  |  |  |  | PtVQ49 | Potri.016G093900 |
| ZmVQ50 | GRMZM2G138370 |  |  |  |  | PtVQ50 | Potri.018G016500 |
| ZmVQ51 | GRMZM2G069169 |  |  |  |  | PtVQ51 | Potri.019G013300 |
| ZmVQ52 | GRMZM2G122447 |  |  |  |  |  |  |
| ZmVQ53 | GRMZM2G333049 |  |  |  |  |  |  |
| ZmVQ54 | GRMZM2G035531 |  |  |  |  |  |  |
| ZmVQ55 | GRMZM2G014839 |  |  |  |  |  |  |
| ZmVQ56 | GRMZM2G129815 |  |  |  |  |  |  |
| ZmVQ57 | GRMZM5G864133 |  |  |  |  |  |  |
| ZmVQ58 | GRMZM2G180262 |  |  |  |  |  |  |
| ZmVQ59 | GRMZM2G060720 |  |  |  |  |  |  |
| ZmVQ60 | GRMZM2G064903 |  |  |  |  |  |  |
| ZmVQ61 | GRMZM2G475276 |  |  |  |  |  |  |

Table S2 Numbers of *VQ* genes and *VQ* genes without introns in different species

| Name | | Species | VQ gene number | Without introns |
| --- | --- | --- | --- | --- |
| Byrophytes | | Moss(Pp) | 25 | 7(28%) |
| Angiosperms | Dicotyledons | Arabidopsis(At) | 34 | 30(88%) |
|  |  | Poplar(Pt) | 51 | 40(78%) |
|  |  | Chinese Cabbage(Br) | 57 | 54(95%) |
|  |  | Soybean(Gm) | 74 | 60(81%) |
|  | Monocotyledons | Rice(Os) | 40 | 37(93%) |
|  |  | Maize(Zm) | 61 | 54(89%) |
|  |  | Grape(Vv) | 18 | 16(89%) |
|  |  | Moso bamboo(Pe) | 29 | 25(86%) |
|  |  | Wheat(Ta) | 65 | 61(94%) |

Table S3 VQ domain types in different species

|  | LTG | FTG | ITG | VIG | LTS | LTD | YTG | LTR | LTV | ATG | LTA |
| --- | --- | --- | --- | --- | --- | --- | --- | --- | --- | --- | --- |
| Arabidopsis(At) | 24 | 5 | 0 | 2 | 1 | 1 | 1 | 0 | 0 | 0 | 0 |
| Poplar(Pt) | 39 | 11 | 0 | 2 | 0 | 0 | 0 | 0 | 0 | 0 | 0 |
| Chinese Cabbage(Br) | 43 | 8 | 0 | 3 | 1 | 0 | 1 | 0 | 1 | 0 | 0 |
| Soybean(Gm) | 55 | 15 | 0 | 2 | 1 | 0 | 0 | 1 | 0 | 0 | 0 |
| Grape(Vv) | 14 | 3 | 0 | 1 | 0 | 0 | 0 | 0 | 0 | 0 | 0 |
| Maize(Zm) | 42 | 8 | 2 | 6 | 0 | 0 | 0 | 0 | 0 | 2 | 1 |
| Rice(Os) | 28 | 7 | 1 | 4 | 0 | 0 | 0 | 0 | 0 | 0 | 0 |
| Moso bamboo(Pe) | 21 | 4 | 3 | 1 | 0 | 0 | 0 | 0 | 0 | 0 | 1 |
| Wheat(Ta) | 52 | 10 | 3 | 0 | 0 | 0 | 0 | 0 | 0 | 0 | 0 |

Table S4Information on 20 conserved motifs of the TaVQ protein family

| MOTIF | Length | Best possible match |
| --- | --- | --- |
| 1 | 41 | GGRRGPVIVYRASPTVVHTDPSEFRALVQRLTGKPSPAPAR |
| 2 | 21 | RAGGLQGPRPQPLSVSKRPHK |
| 3 | 21 | LLSPSSFFFSPTTMQAIRELI |
| 4 | 20 | ENSRRKKIKCEVVEVEEGGF |
| 5 | 15 | DALVLTLGQQQRSPC |
| 6 | 25 | GAAEDRLLLQSIQAAQMPPRPAAYN |
| 7 | 11 | FAELWSDLSPG |
| 8 | 29 | AGQMAAPWCSFPLMGPPAAMHPGLDGHHL |
| 9 | 50 | SAPAGATAGAGKRALPPLPGAPSPYAAAAPAMPPPFASPPSRGEGAGLLL |
| 10 | 50 | LDTPWAHKPATAPAPWPPATWPSSAAPPFENDGJATALARASASAAATTT |
| 11 | 41 | AHDAGAVTVPVVPTPRPPLZEKPRPQPKRLEPAPPLPDDYV |
| 12 | 40 | STGAGASPSPSGLGYFPAGGAPSPPTPYYSDPFNPSPRNL |
| 13 | 50 | RWEDKTSGGAAAATAMPAMADDSSLRLMQELEAMVSAPPAVSSFPTLESW |
| 14 | 11 | QHHHHQHQQQQ |
| 15 | 41 | MGDTGANMGHWAGIYGVGGNGAAEAEGSVVTVSSPTSGGSG |
| 16 | 50 | QNLLGSTQPSSQHMFGGMSHGASRLHEQSPSEFMPGAGSGSMGLTHGGMM |
| 17 | 50 | RGGALKGEAPMVSPWLHQTSDHFLSPGGASALGSPSGFLDIFGPLSSQQQ |
| 18 | 15 | GFLSFLEEDVFQGMI |
| 19 | 15 | PRMDLVGEMCASFLA |
| 20 | 50 | MWNRPHPLSHSRPGALSPLLRVLPRAVAGPPLPSEHDRIRVAALPRMDAT |

Table S5 Transcriptome data for VQ genes

| Gene | J411-4h | J411-6h | J411-10h | HMC21-4h | HMC21-6h | HMC21-10h |
| --- | --- | --- | --- | --- | --- | --- |
| TaVQ1 | 2.976275 | 0.6416393 | 0.8540363 | 0.0930373 | 0.0200477 | 0 |
| TaVQ2 | 0.2035877 | 0.014251 | 0.016983 | 0.0406857 | 0.0343537 | 0.0265607 |
| TaVQ3 | 0.0517697 | 0.013821 | 0.041997 | 0.0399047 | 0 | 0 |
| TaVQ4 | 0.141952 | 0.2900043 | 0.316766 | 0.2155813 | 0.3538523 | 0.778389 |
| TaVQ5 | 0.0631203 | 0.010344 | 0.0869783 | 0.0627393 | 0 | 0 |
| TaVQ6 | 0.0412757 | 0.0415853 | 0.0765583 | 0 | 0.0109623 | 0 |
| TaVQ7 | 0.2034243 | 0.324037 | 0.2382433 | 0.1087323 | 0.5434513 | 0.4534263 |
| TaVQ8 | 0 | 0 | 0 | 0.3098733 | 0.0297947 | 0.031415 |
| TaVQ9 | 0.0034993 | 0 | 0.0029167 | 0 | 0 | 0 |
| TaVQ10 | 0.0435003 | 0.0423773 | 0.0760583 | 0 | 0 | 0 |
| TaVQ11 | 0.3687023 | 0.0178337 | 0.060103 | 0.6615907 | 0.0197223 | 0.131081 |
| TaVQ12 | 0.4406247 | 0.1579423 | 0.2145143 | 0.5430793 | 0.2029477 | 0.041478 |
| TaVQ14 | 0.231753 | 0.0122187 | 0.0452067 | 0.0785007 | 0.0939857 | 0.0211287 |
| TaVQ15 | 0.4744187 | 0.0351557 | 0.260407 | 0.49015 | 0.4230243 | 0.17021 |
| TaVQ16 | 19.584111 | 3.1160873 | 1.409126 | 12.118203 | 2.2277063 | 2.0434733 |
| TaVQ17 | 0.0911387 | 0.040028 | 0 | 0.0368357 | 0 | 0.039929 |
| TaVQ19 | 0.128839 | 0.0214087 | 0.0896787 | 0.106042 | 0.021254 | 0 |
| TaVQ20 | 0 | 0 | 0.024032 | 2.5038927 | 0.9392607 | 2.5938293 |
| TaVQ21 | 0.0518983 | 0.2057433 | 0.1427617 | 0 | 0.0414987 | 0.038199 |
| TaVQ22 | 1.2825463 | 0.969677 | 0.0575263 | 0 | 0 | 0.0700687 |
| TaVQ23 | 0.237939 | 0 | 0 | 0 | 0 | 0 |
| TaVQ24 | 0 | 0 | 0 | 0 | 0 | 0 |
| TaVQ25 | 31.092722 | 10.577266 | 6.1828827 | 3.873434 | 2.1261337 | 3.2225153 |
| TaVQ26 | 0.0449963 | 0.1224243 | 0 | 0 | 0 | 0 |
| TaVQ27 | 0.396513 | 1.1529137 | 0.1142937 | 0.027658 | 0.00755 | 0.024522 |
| TaVQ28 | 1.854342 | 3.2887953 | 0.3362253 | 0.0302643 | 0.021161 | 0.0243517 |
| TaVQ29 | 12.389051 | 3.2571772 | 1.8735101 | 0.2306266 | 0.182781 | 1.4017327 |
| TaVQ30 | 21.974636 | 12.333432 | 4.4214387 | 2.221666 | 1.5009763 | 1.60478 |
| TaVQ31 | 15.878686 | 5.405742 | 0.4237017 | 16.421631 | 4.3134943 | 4.471482 |
| TaVQ32 | 1.2916827 | 0.8719857 | 0.490211 | 1.268079 | 0.574351 | 0.7100963 |
| TaVQ33 | 2.3218697 | 1.8161213 | 1.274912 | 0.8751077 | 0.950627 | 1.2694423 |
| TaVQ34 | 15.335752 | 5.287503 | 2.858194 | 1.1967517 | 1.0953913 | 2.0239677 |
| TaVQ35 | 2.9717493 | 1.280658 | 0.803858 | 7.5602667 | 1.2950017 | 1.8286163 |
| TaVQ36 | 0.5562383 | 0.4202743 | 0.195837 | 0.265821 | 0.1857033 | 0.1514773 |
| TaVQ37 | 0.966398 | 0.5931667 | 0.7986537 | 0.703438 | 0.7988483 | 0.700182 |
| TaVQ38 | 2.2093197 | 1.3144027 | 0.6735993 | 0.357607 | 0.204907 | 1.2386213 |
| TaVQ39 | 0.0214443 | 0 | 0 | 0 | 0 | 0 |
| TaVQ40 | 42.495355 | 2.9280503 | 3.4096167 | 40.160849 | 3.9002523 | 2.7096027 |
| TaVQ41 | 1.4158893 | 0.7652523 | 0.532686 | 1.690973 | 0.7148543 | 0.3656473 |
| TaVQ42 | 0.8598843 | 0.7953673 | 0.8396087 | 0.834481 | 0.775655 | 0.7316077 |
| TaVQ43 | 13.158591 | 7.313306 | 1.7629507 | 0.7457303 | 0.4753833 | 1.213946 |
| TaVQ44 | 16.213773 | 23.072398 | 7.69324 | 1.5752853 | 2.775632 | 4.7739883 |
| TaVQ46 | 0.8402783 | 0.0940893 | 0.057883 | 0.2992037 | 0.016246 | 0.04019 |
| TaVQ47 | 23.910233 | 27.673665 | 11.010195 | 3.80353 | 4.2896393 | 5.3270783 |
| TaVQ48 | 1.2878807 | 0.7198073 | 0.4270627 | 0.533275 | 0.2935693 | 0.362654 |
| TaVQ49 | 6.0934867 | 8.6759193 | 3.2484501 | 0.8813349 | 1.2770255 | 1.0365373 |
| TaVQ50 | 0.9492067 | 0.0555513 | 0 | 0.0825323 | 0.0035997 | 0.0058073 |
| TaVQ51 | 3.8285613 | 1.5355163 | 0.2982257 | 0 | 0.0551127 | 0.160079 |
| TaVQ52 | 17.613559 | 6.0074697 | 1.400376 | 0.3180283 | 0.118971 | 0.7118077 |
| TaVQ53 | 1.166639 | 2.5613673 | 0.2632907 | 0 | 0 | 0.052261 |
| TaVQ54 | 0.0899277 | 0.0307873 | 0.0268767 | 0 | 0.0462657 | 0.0130453 |
| TaVQ55 | 2.9035923 | 2.1713377 | 1.124944 | 0.27333 | 1.0270923 | 1.8925463 |
| TaVQ56 | 1.6356867 | 0.5419437 | 0.1279527 | 0 | 0 | 0.173995 |
| TaVQ57 | 16.767823 | 9.236615 | 2.512072 | 1.3504133 | 0.3656233 | 1.675781 |
| TaVQ58 | 1.789512 | 1.9613387 | 0 | 0 | 0 | 0.062796 |
| TaVQ59 | 0.0738087 | 0.0203983 | 0 | 0 | 0.1308577 | 0.0051733 |
| TaVQ60 | 0.975719 | 0.7646403 | 0.6339437 | 0.735397 | 0.2876457 | 0.551439 |
| TaVQ61 | 3.3358023 | 1.6988607 | 0.0704643 | 0.0012757 | 0.018486 | 0.095102 |
| TaVQ62 | 5.0178593 | 4.4064617 | 0.855188 | 0.3837133 | 0.1358543 | 0.4568247 |
| TaVQ63 | 1.8165833 | 3.6494647 | 0.0419557 | 0 | 0 | 0.0769797 |
| TaVQ64 | 0.2095143 | 0.1384117 | 0.0718653 | 0.0237513 | 0.015323 | 0.0565403 |
| TaVQ65 | 0.941149 | 0.666699 | 0.9073103 | 0.170211 | 0.250874 | 1.5114183 |

Table S6 Microarray data for VQ genes

|  | GSC | GSR | GSE | SR | SC | SL | II | Fba | Pba | Aba | 3-5 DAP C | 22 DAP EM | 22 DAP EN |
| --- | --- | --- | --- | --- | --- | --- | --- | --- | --- | --- | --- | --- | --- |
| TaVQ2 | 1.8132451 | 1.6613362 | 1.4095967 | 1.5505528 | 1.7678408 | 2.2756875 | 2.1924652 | 1.8471612 | 1.7690941 | 2.3540059 | 2.287301481 | 1.726142868 | 2.12255335 |
| TaVQ5 | 1.8132451 | 1.6613362 | 1.4095967 | 1.5505528 | 1.7678408 | 2.2756875 | 2.1924652 | 1.8471612 | 1.7690941 | 2.3540059 | 2.287301481 | 1.726142868 | 2.12255335 |
| TaVQ8 | 1.8132451 | 1.6613362 | 1.4095967 | 1.5505528 | 1.7678408 | 2.2756875 | 2.1924652 | 1.8471612 | 1.7690941 | 2.3540059 | 2.287301481 | 1.726142868 | 2.12255335 |
| TaVQ16 | 0.9398885 | 0.7914801 | 1.0585285 | 0.9344715 | 1.50764 | 1.6393636 | 0.9993321 | 0.9939063 | 0.834891 | 1.9508028 | 0.785279995 | 1.027074944 | 1.871218662 |
| TaVQ31 | 0.9398885 | 0.7914801 | 1.0585285 | 0.9344715 | 1.50764 | 1.6393636 | 0.9993321 | 0.9939063 | 0.834891 | 1.9508028 | 0.785279995 | 1.027074944 | 1.871218662 |
| TaVQ35 | 0.9398885 | 0.7914801 | 1.0585285 | 0.9344715 | 1.50764 | 1.6393636 | 0.9993321 | 0.9939063 | 0.834891 | 1.9508028 | 0.785279995 | 1.027074944 | 1.871218662 |
| TaVQ42 | 1.3366251 | 1.2404161 | 1.5511653 | 1.8636856 | 1.6460474 | 1.3658092 | 1.4303584 | 1.9871208 | 1.8250798 | 1.2884244 | 1.341074277 | 1.594749711 | 1.702725136 |
| TaVQ48 | 1.9781988 | 2.1892657 | 1.8472518 | 2.2894849 | 1.8240889 | 2.2803127 | 1.8743523 | 1.9968647 | 1.955351 | 2.0496056 | 2.022502603 | 2.096607639 | 2.097331364 |
| TaVQ55 | 2.0779115 | 2.2381967 | 1.9868866 | 2.1019841 | 1.1088389 | 2.3757422 | 1.4019528 | 2.2224216 | 2.400912 | 1.1424166 | 1.366447862 | 1.217590092 | 1.389821463 |
| TaVQ60 | 2.0779115 | 2.2381967 | 1.9868866 | 2.1019841 | 1.1088389 | 2.3757422 | 1.4019528 | 2.2224216 | 2.400912 | 1.1424166 | 1.366447862 | 1.217590092 | 1.389821463 |
| TaVQ65 | 2.0779115 | 2.2381967 | 1.9868866 | 2.1019841 | 1.1088389 | 2.3757422 | 1.4019528 | 2.2224216 | 2.400912 | 1.1424166 | 1.366447862 | 1.217590092 | 1.389821463 |

Table S7 Promoter analysis of the TaVQ protein family

| Gene | biotic stress | | | | | | | | abiotic stress | | |
| --- | --- | --- | --- | --- | --- | --- | --- | --- | --- | --- | --- |
|  | ABA | MeJA | | Auxin | | SA | GA | | Drought | Low Temp | Defense |
|  | ABRE | CGTCA-motif | TGACG-motif | TGA-element | AuxRR-core | TCA-element | GARE-motif | P-box | MBS | LTR | TC-rich repeats |
| TaVQ1 | 3 | 2 | 2 |  | 1 |  |  |  | 1 |  | 1 |
| TaVQ2 | 4 | 3 | 3 |  |  |  | 1 |  |  |  | 1 |
| TaVQ3 | 5 | 3 | 3 |  | 1 | 1 |  |  | 2 |  |  |
| TaVQ4 | 2 | 1 | 1 |  |  | 1 |  |  |  | 1 |  |
| TaVQ5 | 3 | 1 | 1 | 1 |  | 1 |  |  | 1 |  |  |
| TaVQ6 | 4 | 3 | 3 | 1 |  | 1 |  |  |  |  |  |
| TaVQ7 | 5 | 1 | 1 |  |  | 1 |  |  |  | 2 |  |
| TaVQ8 | 6 | 3 | 3 | 1 |  |  |  |  | 1 |  |  |
| TaVQ9 | 7 | 4 | 4 |  | 1 |  |  |  | 1 |  |  |
| TaVQ10 | 3 | 1 | 1 |  |  |  |  |  |  |  |  |
| TaVQ11 | 2 | 3 | 3 |  |  | 1 |  |  |  | 1 |  |
| TaVQ12 |  | 1 | 1 |  |  |  |  |  | 1 |  |  |
| TaVQ13 | 1 | 3 | 3 |  |  |  |  |  | 1 | 1 | 1 |
| TaVQ14 |  | 2 | 2 | 2 | 1 | 1 |  |  |  |  |  |
| TaVQ15 | 1 |  |  |  |  |  |  |  |  |  | 1 |
| TaVQ16 | 11 | 4 | 4 | 1 |  |  |  |  |  | 2 |  |
| TaVQ17 | 3 | 2 | 2 | 1 |  | 1 |  |  | 2 | 1 |  |
| TaVQ18 |  | 1 | 1 | 1 |  |  |  |  |  |  |  |
| TaVQ19 | 4 | 2 | 2 | 1 |  |  |  |  | 1 | 1 | 1 |
| TaVQ20 | 2 | 1 | 1 | 1 |  |  |  |  |  |  |  |
| TaVQ21 | 2 | 2 | 2 |  |  |  |  |  | 1 | 1 |  |
| TaVQ22 |  | 3 | 3 | 1 |  |  | 1 |  | 1 |  |  |
| TaVQ23 | 1 | 3 | 3 | 1 |  |  |  |  |  |  |  |
| TaVQ24 | 4 | 3 | 3 | 2 |  |  | 1 |  | 1 | 1 |  |
| TaVQ25 | 14 | 3 | 3 |  |  |  |  |  |  |  |  |
| TaVQ26 | 1 | 3 | 3 |  | 1 |  | 1 |  |  |  |  |
| TaVQ27 | 3 | 3 | 3 | 2 |  |  | 1 | 1 |  |  |  |
| TaVQ28 |  | 3 | 3 | 1 |  |  | 1 |  |  |  |  |
| TaVQ29 | 10 | 1 | 1 |  |  |  |  |  |  |  |  |
| TaVQ30 | 9 | 2 | 2 |  |  |  |  |  |  |  | 1 |
| TaVQ31 | 5 | 4 | 4 |  |  | 1 |  | 2 | 1 |  | 1 |
| TaVQ32 | 2 | 3 | 3 |  |  | 3 |  |  |  | 1 | 1 |
| TaVQ33 |  | 1 | 1 | 2 |  | 2 |  | 1 | 3 |  |  |
| TaVQ34 | 1 | 1 | 1 |  | 2 | 1 |  |  |  |  |  |
| TaVQ35 | 10 | 1 | 1 |  |  |  | 1 |  |  | 1 |  |
| TaVQ36 | 2 | 1 | 1 |  |  |  | 1 |  | 1 |  |  |
| TaVQ37 | 3 |  |  | 1 |  |  |  |  | 1 |  |  |
| TaVQ38 | 1 |  |  | 1 |  |  |  | 1 |  | 1 | 1 |
| TaVQ39 |  | 2 | 2 |  |  | 1 | 1 | 1 |  |  |  |
| TaVQ40 | 12 | 2 | 2 |  |  |  |  |  |  |  |  |
| TaVQ41 | 3 | 1 | 1 |  |  |  | 1 |  |  |  | 1 |
| TaVQ42 | 2 | 2 | 2 |  |  |  |  |  |  | 1 |  |
| TaVQ43 | 4 | 1 | 1 |  |  |  |  | 1 |  |  |  |
| TaVQ44 | 8 |  |  | 1 |  | 1 | 1 |  |  |  |  |
| TaVQ45 | 2 | 1 | 1 | 1 |  |  |  |  |  |  | 1 |
| TaVQ46 | 5 | 2 | 2 | 1 |  |  |  |  | 1 | 1 |  |
| TaVQ47 | 7 |  |  | 1 |  | 2 |  |  |  |  |  |
| TaVQ48 | 4 | 2 | 2 |  |  | 1 |  |  | 3 | 1 |  |
| TaVQ49 | 7 | 4 | 4 | 1 |  | 1 |  |  |  |  |  |
| TaVQ50 | 5 | 1 | 1 | 1 |  | 1 |  |  | 2 | 1 |  |
| TaVQ51 | 4 | 4 | 4 | 1 |  |  |  | 1 |  |  | 1 |
| TaVQ52 | 5 | 7 | 7 |  |  |  |  |  | 1 |  |  |
| TaVQ53 | 5 | 1 | 1 |  |  |  |  | 1 |  |  |  |
| TaVQ54 | 3 | 4 | 4 | 1 |  |  | 1 |  |  |  |  |
| TaVQ55 | 9 | 2 | 2 | 1 |  |  |  |  |  |  |  |
| TaVQ56 | 4 | 3 | 3 | 2 |  |  |  | 2 |  |  |  |
| TaVQ57 | 3 | 2 | 2 | 1 |  |  |  |  |  | 1 |  |
| TaVQ58 | 4 | 2 | 2 |  |  |  | 1 | 1 |  |  |  |
| TaVQ59 | 3 |  |  | 2 |  |  |  | 1 |  |  |  |
| TaVQ60 | 6 | 3 | 3 | 1 |  |  |  |  | 1 | 1 |  |
| TaVQ61 | 5 | 3 | 3 | 3 |  |  |  | 1 |  | 1 |  |
| TaVQ62 | 2 | 1 | 1 |  |  | 1 |  |  |  | 1 |  |
| TaVQ63 | 10 | 5 | 5 |  |  |  | 1 |  |  | 2 |  |
| TaVQ64 | 5 | 2 | 2 | 1 |  | 1 |  |  | 2 | 1 | 1 |
| TaVQ65 | 11 | 4 | 4 | 1 |  |  |  |  | 1 | 1 |  |

Table S8 Gene ontology (GO) annotations of TaVQ proteins

| Name | GO:0005515 | GO:0005634 | GO:0006952 | GO:0043433 | GO:0051245 | GO:0003674 | GO:0010337 | GO:0005516 | GO:0008150 | GO:0009870 | GO:0005739 | GO:0009507 | GO:0010224 | GO:0009414 | GO:0009651 |
| --- | --- | --- | --- | --- | --- | --- | --- | --- | --- | --- | --- | --- | --- | --- | --- |
| TaVQ1 | √ | √ | √ | √ | √ |  |  |  |  |  |  |  |  |  |  |
| TaVQ2 |  | √ | √ |  |  | √ |  |  |  |  |  |  |  |  |  |
| TaVQ3 |  |  |  |  |  |  |  |  |  |  |  |  |  |  |  |
| TaVQ4 | √ | √ | √ |  |  |  |  |  |  | √ |  |  |  |  |  |
| TaVQ5 |  | √ | √ |  |  | √ |  |  |  |  |  |  |  |  |  |
| TaVQ6 |  |  |  |  |  |  |  |  |  |  |  |  |  |  |  |
| TaVQ7 | √ | √ | √ |  |  |  |  |  |  | √ |  |  |  |  |  |
| TaVQ8 |  | √ | √ |  |  | √ |  |  |  |  |  |  |  |  |  |
| TaVQ9 |  |  |  |  |  |  |  |  |  |  |  |  |  |  |  |
| TaVQ10 |  | √ |  |  |  | √ |  |  | √ |  |  |  |  |  |  |
| TaVQ11 |  | √ |  |  |  | √ |  |  | √ |  |  |  |  |  |  |
| TaVQ12 |  |  |  |  |  |  |  |  |  |  |  |  |  |  |  |
| TaVQ13 |  |  |  |  |  |  |  |  |  |  |  |  |  |  |  |
| TaVQ14 | √ | √ |  |  |  |  | √ | √ |  |  |  |  |  | √ | √ |
| TaVQ15 |  |  |  |  |  |  |  |  |  |  |  |  |  |  |  |
| TaVQ16 |  |  |  |  |  |  |  |  |  |  |  |  |  |  |  |
| TaVQ17 |  | √ |  |  |  | √ |  |  | √ |  |  |  |  |  |  |
| TaVQ18 |  |  |  |  |  |  |  |  |  |  |  |  |  |  |  |
| TaVQ19 |  |  |  |  |  |  |  |  |  |  |  |  |  |  |  |
| TaVQ20 | √ | √ | √ | √ | √ |  |  |  |  |  |  |  |  |  |  |
| TaVQ21 |  |  |  |  |  |  |  |  |  |  |  |  |  |  |  |
| TaVQ22 |  |  |  |  |  |  |  |  |  |  |  |  |  |  |  |
| TaVQ23 |  |  |  |  |  |  |  |  |  |  |  |  |  |  |  |
| TaVQ24 |  |  |  |  |  |  |  |  |  |  |  |  |  |  |  |
| TaVQ25 |  |  |  |  |  |  |  |  |  |  |  |  |  |  |  |
| TaVQ26 |  |  |  |  |  |  |  |  |  |  |  |  |  |  |  |
| TaVQ27 |  |  |  |  |  |  |  |  |  |  |  |  |  |  |  |
| TaVQ28 |  |  |  |  |  |  |  |  |  |  |  |  |  |  |  |
| TaVQ29 |  |  |  |  |  |  |  |  |  |  |  |  |  |  |  |
| TaVQ30 |  |  |  |  |  |  |  |  |  |  |  |  |  |  |  |
| TaVQ31 |  | √ |  |  |  | √ |  |  |  |  |  |  | √ |  |  |
| TaVQ32 |  | √ | √ |  |  | √ |  |  |  |  |  |  |  |  |  |
| TaVQ33 |  |  |  |  |  |  |  |  |  |  |  |  |  |  |  |
| TaVQ34 |  | √ |  |  |  | √ |  |  | √ |  |  | √ |  |  |  |
| TaVQ35 |  | √ |  |  |  | √ |  |  |  |  |  |  | √ |  |  |
| TaVQ36 |  | √ | √ |  |  | √ |  |  |  |  |  |  |  |  |  |
| TaVQ37 |  | √ |  |  |  | √ |  |  | √ |  |  |  |  |  |  |
| TaVQ38 |  | √ |  |  |  | √ |  |  | √ |  |  | √ |  |  |  |
| TaVQ39 |  |  |  |  |  |  |  |  |  |  |  |  |  |  |  |
| TaVQ40 |  | √ |  |  |  | √ |  |  |  |  |  |  | √ |  |  |
| TaVQ41 |  | √ | √ |  |  | √ |  |  |  |  |  |  |  |  |  |
| TaVQ42 |  |  |  |  |  |  |  |  |  |  |  |  |  |  |  |
| TaVQ43 |  | √ |  |  |  | √ |  |  | √ |  |  | √ |  |  |  |
| TaVQ44 |  | √ |  |  |  | √ |  |  | √ |  |  |  |  |  |  |
| TaVQ45 |  |  |  |  |  |  |  |  |  |  |  |  |  |  |  |
| TaVQ46 |  |  |  |  |  |  |  |  |  |  |  |  |  |  |  |
| TaVQ47 |  | √ |  |  |  | √ |  |  | √ |  |  |  |  |  |  |
| TaVQ48 |  |  |  |  |  |  |  |  |  |  |  |  |  |  |  |
| TaVQ49 |  |  |  |  |  |  |  |  |  |  |  |  |  |  |  |
| TaVQ50 |  |  |  |  |  |  |  |  |  |  |  |  |  |  |  |
| TaVQ51 | √ | √ | √ |  |  |  |  |  |  | √ |  |  |  |  |  |
| TaVQ52 |  |  |  |  |  |  |  |  |  |  |  |  |  |  |  |
| TaVQ53 |  |  |  |  |  |  |  |  |  |  |  |  |  |  |  |
| TaVQ54 |  | √ |  |  |  | √ |  |  | √ |  | √ |  |  |  |  |
| TaVQ55 |  | √ |  |  |  | √ |  |  | √ |  |  |  |  |  |  |
| TaVQ56 | √ | √ | √ |  |  |  |  |  |  | √ |  |  |  |  |  |
| TaVQ57 |  |  |  |  |  |  |  |  |  |  |  |  |  |  |  |
| TaVQ58 |  |  |  |  |  |  |  |  |  |  |  |  |  |  |  |
| TaVQ59 |  | √ |  |  |  | √ |  |  | √ |  |  |  |  |  |  |
| TaVQ60 |  | √ |  |  |  | √ |  |  | √ |  |  |  |  |  |  |
| TaVQ61 | √ | √ | √ |  |  |  |  |  |  | √ |  |  |  |  |  |
| TaVQ62 |  |  |  |  |  |  |  |  |  |  |  |  |  |  |  |
| TaVQ63 |  |  |  |  |  |  |  |  |  |  |  |  |  |  |  |
| TaVQ64 |  | √ |  |  |  | √ |  |  | √ |  | √ |  |  |  |  |
| TaVQ65 |  | √ |  |  |  | √ |  |  | √ |  |  |  |  |  |  |

GO definitions for the *TaVQ* genes

| Accession | Ontology | Name | Definition |
| --- | --- | --- | --- |
| GO:0005515 | molecular_function | protein binding | Interacting selectively and non-covalently with any protein or protein complex (a complex of two or more proteins that may include other nonprotein molecules). |
| GO:0005634 | cellular_component | nucleus | A membrane-bounded organelle of eukaryotic cells in which chromosomes are housed and replicated. In most cells, the nucleus contains all of the cell's chromosomes except the organellar chromosomes, and is the site of RNA synthesis and processing. In some species, or in specialized cell types, RNA metabolism or DNA replication may be absent. |
| GO:0006952 | biological_process | defense response | Reactions, triggered in response to the presence of a foreign body or the occurrence of an injury, which result in restriction of damage to the organism attacked or prevention/recovery from the infection caused by the attack. |
| GO:0043433 | biological_process | negative regulation of DNA-binding transcription factor activity | Any process that stops, prevents, or reduces the frequency, rate or extent of the activity of a transcription factor, any factor involved in the initiation or regulation of transcription |
| GO:0051245 | biological_process | negative regulation of cellular defense response | Any process that stops, prevents, or reduces the rate of the cellular defense response. |
| GO:0003674 | molecular_function | molecular_function | A molecular process that can be carried out by the action of a single macromolecular machine, usually via direct physical interactions with other molecular entities. Function in this sense denotes an action, or activity, that a gene product (or a complex) performs. These actions are described from two distinct but related perspectives: (1) biochemical activity, and (2) role as a component in a larger system/process. |
| GO:0010337 | biological_process | regulation of salicylic acid metabolic process | Any process that modulates the frequency, rate or extent of the chemical reactions and pathways involving salicylic acid |
| GO:0005516 | molecular_function | calmodulin binding | Interacting selectively and non-covalently with calmodulin, a calcium-binding protein with many roles, both in the calcium-bound and calcium-free states. |
| GO:0008150 | biological_process | biological_process | A biological process represents a specific objective that the organism is genetically programmed to achieve. Biological processes are often described by their outcome or ending state, e.g., the biological process of cell division results in the creation of two daughter cells (a divided cell) from a single parent cell. A biological process is accomplished by a particular set of molecular functions carried out by specific gene products (or macromolecular complexes), often in a highly regulated manner and in a particular temporal sequence. |
| GO:0009870 | biological_process | defense response signaling pathway, resistance gene-dependent | A series of molecular signals generated as a consequence of a pathogen or microbial effector binding to a plant 'resistance-gene' receptor to activate a plant immune response, usually plant-type hypersensitive response. |
| GO:0005739 | cellular_component | mitochondrion | A semiautonomous, self replicating organelle that occurs in varying numbers, shapes, and sizes in the cytoplasm of virtually all eukaryotic cells. It is notably the site of tissue respiration. |
| GO:0009507 | cellular_component | chloroplast | A chlorophyll-containing plastid with thylakoids organized into grana and frets, or stroma thylakoids, and embedded in a stroma. |
| GO:0010224 | biological_process | response to UV-B | Any process that results in a change in state or activity of a cell or an organism (in terms of movement, secretion, enzyme production, gene expression, etc.) as a result of a UV-B radiation stimulus. UV-B radiation (UV-B light) spans the wavelengths 280 to 315 nm. |
| GO:0009414 | biological_process | response to water deprivation | Any process that results in a change in state or activity of a cell or an organism (in terms of movement, secretion, enzyme production, gene expression, etc.) as a result of a water deprivation stimulus, prolonged deprivation of water. |
| GO:0009651 | biological_process | response to salt stress | Any process that results in a change in state or activity of a cell or an organism (in terms of movement, secretion, enzyme production, gene expression, etc.) as a result of a stimulus indicating an increase or decrease in the concentration of salt (particularly but not exclusively sodium and chloride ions) in the environment. |

TableS9 Subcellular localization of *TaVQ*s predicted by WOLF PSORT

| Name | Position | Name | Position | Name | Position | Name | Position | Name | Position |
| --- | --- | --- | --- | --- | --- | --- | --- | --- | --- |
| TaVQ1 | Periplasmic | TaVQ16 | Periplasmic | TaVQ31 | Periplasmic | TaVQ46 | Periplasmic | TaVQ61 | Periplasmic |
| TaVQ2 | Periplasmic | TaVQ17 | Extracellular | TaVQ32 | Periplasmic | TaVQ47 | Extracellular | TaVQ62 | Periplasmic |
| TaVQ3 | Periplasmic | TaVQ18 | Cytoplasmic | TaVQ33 | Extracellular | TaVQ48 | Periplasmic | TaVQ63 | Periplasmic |
| TaVQ4 | Periplasmic | TaVQ19 | Periplasmic | TaVQ34 | Periplasmic | TaVQ49 | Extracellular | TaVQ64 | Periplasmic |
| TaVQ5 | Periplasmic | TaVQ20 | Periplasmic | TaVQ35 | Periplasmic | TaVQ50 | Periplasmic | TaVQ65 | Extracellular |
| TaVQ6 | Periplasmic | TaVQ21 | Periplasmic | TaVQ36 | Periplasmic | TaVQ51 | Periplasmic |  |  |
| TaVQ7 | Periplasmic | TaVQ22 | Periplasmic | TaVQ37 | Extracellular | TaVQ52 | Periplasmic |  |  |
| TaVQ8 | Periplasmic | TaVQ23 | Periplasmic | TaVQ38 | Periplasmic | TaVQ53 | Periplasmic |  |  |
| TaVQ9 | Periplasmic | TaVQ24 | Periplasmic | TaVQ39 | Periplasmic | TaVQ54 | Periplasmic |  |  |
| TaVQ10 | Extracellular | TaVQ25 | Extracellular | TaVQ40 | Periplasmic | TaVQ55 | Extracellular |  |  |
| TaVQ11 | Periplasmic | TaVQ26 | Periplasmic | TaVQ41 | Periplasmic | TaVQ56 | Periplasmic |  |  |
| TaVQ12 | Extracellular | TaVQ27 | Periplasmic | TaVQ42 | Extracellular | TaVQ57 | Periplasmic |  |  |
| TaVQ13 | Cytoplasmic | TaVQ28 | Periplasmic | TaVQ43 | Periplasmic | TaVQ58 | Periplasmic |  |  |
| TaVQ14 | Periplasmic | TaVQ29 | Extracellular | TaVQ44 | Extracellular | TaVQ59 | Cytoplasmic |  |  |
| TaVQ15 | Extracellular | TaVQ30 | Periplasmic | TaVQ45 | Periplasmic | TaVQ60 | Extracellular |  |  |

Table S10 qRT-PCR primers for *TaVQ* genes

| Name | Primers | Name | Primers |
| --- | --- | --- | --- |
| TaActin | F:CTTGTATGCCAGCGGTCGAAC | TaVQ33 | F:ACCACAACAGCGTCTGCCAATC |
|  | R:CTCATAATCAAGGGCCACG |  | R:GTGGAGCCGAGGAGGTTCTGG |
| TaVQ1 | F:CCGAGTCTCACGCCATCAAGAAG | TaVQ34 | F:TCCAGGCCGTTGACCAAGAAGG |
|  | R:GGCTGGCCTTGCTGCTTCTC |  | R:GCTGCACGACGGTCATGAACTC |
| TaVQ2 | F:GCGGAGGTGAAGGAGGAGGAC | TaVQ35 | F:ATGAGGAGGCCGTCCGTGAC |
|  | R:CTGGAAGAAGGCGTCGTTCGTC |  | R:GCTCCACGCGGTACACCTTG |
| TaVQ3 | F:CCACCATCACCACCACCAACAC | TaVQ36 | F:GTTCCAGCCGCAGGAATCGC |
|  | R:CTCTCCACCTCCTCCTGGCTTATC |  | R:CGTCCAGGTCCAGCCAGGAG |
| TaVQ4 | F:CCGAGTCGCACGCCATCAAG | TaVQ37 | F:GCAGGACCACAGCAGCAACTAC |
|  | R:GGCTGGCCTTGCTGCTTCTC |  | R:CATGAACTCCGACGGCGATTGG |
| TaVQ5 | F:GCGGAGGTGAAGGAGGAGGAG | TaVQ38 | F:AGGGAGCTGGAGTAGGAGAC |
|  | R:CTGGAAGAAGGCGTCGTTCGTC |  | R:AAAGCTCCTGGATGGACTGC |
| TaVQ6 | F:CCGAGTCGCACGCCATCAAG | TaVQ39 | F:GTGCGCCGTGTCGTCATGATC |
|  | R:GGCTGGCCTTGCTGCTTCTC |  | R:AGGTCTCGTATCGCCTGCATGG |
| TaVQ7 | F:CCGAGTCGCACGCCATCAAG | TaVQ40 | F:AGCACCAGCACACACATCA |
|  | R:GGCTGGCCTTGCTGCTTCTC |  | R:TACATGAGATGGTGGCCGTC |
| TaVQ8 | F:GCGGAGGTGAAGGAGGAGGAG | TaVQ41 | F:TCATCGTCGGAGGACCGTCAG |
|  | R:CTGGAAGAAGGCGTCGTTCGTC |  | R:TTGCTGGTGGCGGTTGTTGG |
| TaVQ9 | F:CCATCACCACCACCACCAACAC | TaVQ42 | F:ACCACAACAGCGTCTGCCAATC |
|  | R:CTCTCCACCTCCTCCTGGCTTATC |  | R:AGGAGGTTCTGGAACGACAGGTAG |
| TaVQ10 | F:CGAGGAGCAACGCTATGCCAAG | TaVQ43 | F:CCGGAGCGCCTATGGAGGAG |
|  | R:TCCGCCGATGCCGAAGTCTC |  | R:CCTGGATGGACTGCATGGTGTTG |
| TaVQ11 | F:TGCAGGAGATCACCGGCTACG | TaVQ44 | F:GCAGCAGCAGCATCAGCAGAG |
|  | R:CAAGAAGCAGGCGGACGTGTC |  | R:CTGTCCTCCGCCGACGACTC |
| TaVQ12 | F:GCCGATCAAGGTGGTGTACATCTC | TaVQ45 | F:CAGCGCCAAGTACCAGCCATG |
|  | R:GCCGTCGTCGGTATTGTACTTGG |  | R:GACACGTCGGTCGCCACAAC |
| TaVQ13 | F:GGAGGCACCACCACCAGACC | TaVQ46 | F:CAGCGCCAAGTACCAGCCATG |
|  | R:CCACCTCGCACTTGATCTTCTTCC |  | R:ACGCCGAGTGCTTCCTCCTG |
| TaVQ14 | F:GCAATACGCCGCCTGGTTCTC | TaVQ47 | F:AGCGTGGTGACGGTGTCTAGC |
|  | R:GTGGTGGTGGAGGAGGAGGAAG |  | R:GGAAGTTGGTGGTGTCGGTGTTG |
| TaVQ15 | F:GCCGATCAAGGTGGTGTACATCTC | TaVQ48 | F:GCGGCGCGTATCATGGAGTC |
|  | R:GCCGTCGTCGGTATTGTACTTGG |  | R:ATGGCGCGGAAGTTGGACAC |
| TaVQ16 | F:CAAGGTGTACCGCGTGGAG | TaVQ49 | F:CGCAGCAGCAGCAGCAGTAC |
|  | R:GCGTCCTCAACACCTCCG |  | R:CTGTCCTCCGCCGACGACTC |
| TaVQ17 | F:GCGAGAGGATCAGTTGCTGCTG | TaVQ50 | F:GCGCCAAGTACCAGCCATGC |
|  | R:CGCCGCCTAAGGAAGAGAAGAAC |  | R:GCGCGGAAGTTGGACACGTC |
| TaVQ18 | F:CACCACCAGAACGCCGATGC | TaVQ51 | F:CACAAGGACTCGCACAGGATCAAG |
|  | R:CACCTTCACCACCTCGCACTTG |  | R:GCTGCACGACGGACATGAACTC |
| TaVQ19 | F:CTCCACCACCACCACCTCCTC | TaVQ52 | F:GGCGGCGTGAAGGTCAAGTTC |
|  | R:GCGGTGATGTACGTGGTCTGC |  | R:GCGGCGGCATCTTCGAGTAC |
| TaVQ20 | F:TCCTCGTCCTCCTCCTCCTACTC | TaVQ53 | F:AGCAGCAGCAGCAGCAGAAC |
|  | R:TTGGTCATACATGCTCTGGAACGG |  | R:GCTGAGGTCGTGGAACAGGAAC |
| TaVQ21 | F:GTCGTCGTCGTCGTCCAAGC | TaVQ54 | F:CAAGTGCGAGGTGGTGAAGGTG |
|  | R:CGTGCGCTCGTAGACGATCAC |  | R:AGCAGGTCAGGAGCCATCATCC |
| TaVQ22 | F:CCGTGCGGTGGTGATCATCATC | TaVQ55 | F:AACACGGACGCCAGCAACTTC |
|  | R:CTCTCGTATCGCCTGCATGGTG |  | R:TGGAGGTGGTGGTGGTCGAAG |
| TaVQ23 | F:GTTCATGGCCGTCGTGCAGAG | TaVQ56 | F:AGGACTCGCACAGGATCAGGAAG |
|  | R:CTGCTGCTGGCTAAGCGTGAG |  | R:GCTGCACGACGGACATGAACTC |
| TaVQ24 | F:GCCGTGTGGTGCTCATCATCC | TaVQ57 | F:GGCGGCGTGAAGGTCAAGTTC |
|  | R:CGTATCGCCTGCATGGTGGTC |  | R:GCGGCGGCATCTTGGAGTAC |
| TaVQ25 | F:GGCAGCACAACGGCATCTCC | TaVQ58 | F:CACCACCACGAGCTACGACAAC |
|  | R:GCTGGCGGTGAGCATCATGG |  | R:GGCTTCCGGTTCTGCTGCTG |
| TaVQ26 | F:GTTCATGGCCGTCGTGCAGAG | TaVQ59 | F:ACGCCGCTATCGCCTGTAGAG |
|  | R:GGACAGCAGCGGTGATGATGC |  | R:CACCTTCACCACCTCGCACTTG |
| TaVQ27 | F:CGCCGTGTGGTGGTGATCATC | TaVQ60 | F:AACACGGACGCCAGCAACTTC |
|  | R:CGTATCGCCTGCATGGTGGTC |  | R:TGGAGGTGGTGGTGGTCGAAG |
| TaVQ28 | F:TGGTTCTCACGCTTAGCCAACAG | TaVQ61 | F:AGGACTCGCACAAGATCAAGAAGC |
|  | R:CGTATCGCCTGCATGGTGGTC |  | R:GGAGTGAGGAGGATGACGAGGAG |
| TaVQ29 | F:GGCAGCACAACGGCATCTCC | TaVQ62 | F:GGCGGCGTGAAGGTCAAGTTC |
|  | R:GCTGGCGGTGAGCATCATGG |  | R:GCGGCGGCATCTTGGAGTAC |
| TaVQ30 | F:GCCGTGTGGTGCTCATCATCC | TaVQ63 | F:AGCAGCAGCAGCAGAACGTG |
|  | R:CGTATCGCCTGCATGGTGGTC |  | R:GCTGAGGTCGTGGAACAGGAAC |
| TaVQ31 | F:TAGCCAGGCAGCACCAGCAC | TaVQ64 | F:CCGCAGCCGCAGAAGAAGAG |
|  | R:CACCACGGCGCGTACATCTG |  | R:CACCTTCACCACCTCGCACTTG |
| TaVQ32 | F:TACGCCGCCATCGAGAGGTC | TaVQ65 | F:AACACGGACGCCAGCAACTTC |
|  | R:CCATCCAGGTCCAGCCAGGAG |  | R:TGGTGGAGGTGGTGGTCGAAG |

TableS11 Data of seed germination index (GI) of six wheat varieties

|  | HMC21 | | | Average GI | SNTT | | | Average GI | YXM | | | Average GI |
| --- | --- | --- | --- | --- | --- | --- | --- | --- | --- | --- | --- | --- |
| 0h | 0 | 0 | 0 | 0 | 0 | 0 | 0 | 0 | 0 | 0 | 0 | 0 |
| 6h | 0 | 0 | 0 |  | 0 | 0 | 0 |  | 0 | 0 | 0 |  |
| 10h | 0 | 0 | 0 |  | 0 | 0 | 0 |  | 0 | 0 | 0 |  |
| 1d | 0 | 0 | 0 | 0.04 | 0 | 0 | 0 | 0.06 | 0 | 0 | 0 | 0.03 |
| 2d | 1 | 1 | 0 |  | 1 | 0 | 2 |  | 0 | 0 | 0 |  |
| 3d | 4 | 5 | 6 |  | 9 | 7 | 7 |  | 4 | 5 | 4 |  |
|  |  |  |  |  |  |  |  |  |  |  |  |  |
|  | J411 | | | Average GI | ZM895 | | | Average GI | ZY9507 | | | Average GI |
| 0h | 0 | 0 | 0 | 0.33 | 0 | 0 | 0 | 0.31 | 0 | 0 | 0 | 0.41 |
| 6h | 20 | 18 | 19 |  | 21 | 19 | 20 |  | 30 | 28 | 29 |  |
| 10h | 30 | 28 | 31 |  | 28 | 26 | 27 |  | 35 | 34 | 36 |  |
| 1d | 39 | 39 | 39 | 0.89 | 31 | 30 | 31 | 0.81 | 40 | 41 | 40 | 0.90 |
| 2d | 48 | 49 | 47 |  | 42 | 43 | 41 |  | 47 | 45 | 45 |  |
| 3d | 48 | 50 | 49 |  | 47 | 48 | 49 |  | 50 | 47 | 50 |  |

Table S12 Data of seed germination rate (GR) of six wheat varieties

|  | HMC21 | | | Average GR | SNTT | | | Average GR | YXM | | | Average GR |
| --- | --- | --- | --- | --- | --- | --- | --- | --- | --- | --- | --- | --- |
| 1d | 0.00% | 0.00% | 0.00% | 0.00% | 0.00% | 0.00% | 0.00% | 0.00% | 0.00% | 0.00% | 0.00% | 0.00% |
| 2d | 2.00% | 2.00% | 0.00% | 1.30% | 2.00% | 0.00% | 4.00% | 2.00% | 0.00% | 0.00% | 0.00% | 0.00% |
| 3d | 8.00% | 10.00% | 12.00% | 10.00% | 16.00% | 15.00% | 17.00% | 16.00% | 8.00% | 10.00% | 8.00% | 9.00% |
|  |  |  |  |  |  |  |  |  |  |  |  |  |
|  | J411 | | | Average GR | ZM895 | | | Average GR | ZY9507 | | | Average GR |
| 1d | 78.00% | 78.00% | 78.00% | 78.00% | 62.00% | 60.00% | 62.00% | 61.33% | 80.00% | 82.00% | 80.00% | 80.66% |
| 2d | 96.00% | 98.00% | 94.00% | 96.00% | 84.00% | 86.00% | 82.00% | 84.00% | 94.00% | 90.00% | 90.00% | 92.00% |
| 3d | 96.00% | 100.00% | 98.00% | 98.00% | 94.00% | 96.00% | 98.00% | 96.00% | 100.00% | 94.00% | 100.00% | 98.00% |
